# Integration of intraoperative data in interpretable machine learning models to predict postoperative AKI in noncardiac surgery patients

**DOI:** 10.1093/jamiaopen/ooag092

**Published:** 2026-06-20

**Authors:** Justin Do, Karan H Shah, Melissa Chen Xu, Andrew Hyunwoo Kim, Vivaswat Suresh, Nidhir Guggilla, Michael Li, Rishi Kothari

**Affiliations:** Sidney Kimmel Medical College, Thomas Jefferson University, Philadelphia, PA 19107, United States; Sidney Kimmel Medical College, Thomas Jefferson University, Philadelphia, PA 19107, United States; Sidney Kimmel Medical College, Thomas Jefferson University, Philadelphia, PA 19107, United States; College of Computing, Data Science, and Society, University of California, Berkeley, Berkeley, CA 94720, United States; Department of Computer Science and Engineering, University of California, San Diego, CA 92093, United States; Department of Electrical Engineering and Computer Sciences, University of California, Berkeley, Berkeley, CA 94720, United States; Center for Digital Health & Data Science, Thomas Jefferson University, Philadelphia, PA 19107, United States; Department of Anesthesiology and Perioperative Medicine, Thomas Jefferson University, Philadelphia, PA 19107, United States

**Keywords:** Machine learning, acute kidney injury, LSTM, time-series, SHAP

## Abstract

**Objectives:**

We aimed to (1) quantify changes in discrimination when adding intraoperative data to preoperative data and (2) compare tabular machine learning with feature engineering against a time-aware LSTM-based model.

**Materials and Methods:**

Retrospective cohort of 46 204 adults undergoing 57 055 eligible noncardiac surgery in the INSPIRE database. We extracted 38 preoperative and 49 intraoperative variables; acute kidney injury (AKI) was defined by KDIGO serum creatinine criteria and modeled as stage 2/3 postoperative AKI. Models were trained on preoperative-only and combined pre- and intraoperative data. Intraoperative series were summarized using eight statistical features for tabular models or integrated directly using an MLP+LSTM architecture.

**Results:**

GBT with combined features achieved the highest AUROC (0.896, 95% CI, 0.878-0.914), followed by combined AutoGluon (0.893, 95% CI, 0.877-0.909) and preoperative-only GBT (0.891, 95% CI, 0.871-0.910). ASA-PS ≥3 (AUROC 0.723, 95% CI, 0.700-0.746) and adapted GS-AKI (AUROC 0.719, 95% CI, 0.700-0.739) underperformed machine-learning models. The hybrid MLP+LSTM model did not outperform simpler tabular models (AUROC 0.870, 95% CI, 0.848-0.892).

**Discussion:**

The small gain from adding low-frequency intraoperative summaries suggests that most discriminative information for stage 2/3 postoperative AKI was available before surgery.

**Conclusion:**

Preoperative tabular ML models provided excellent prediction of postoperative AKI, and added limited incremental discrimination at the available sampling frequency. Future work should evaluate whether higher-frequency intraoperative signals better leverage time-aware architectures.

## Background and significance

Postoperative acute kidney injury (AKI) complicates 12% of surgical procedures and has been associated with worse patient outcomes (such as increased length of stay, readmission rate, development of end-stage renal disease, and mortality).^1^ This complication increases healthcare utilization and thus also increases the total cost of surgical hospitalization by 1.38- to 3-fold for patients undergoing non-cardiac surgery.^2^ Early detection of AKI can improve patient outcomes by encouraging healthcare teams to alter clinical care accordingly. This has led to attempts to identify high-risk patients; however, despite methods like scoring systems or biomarkers, AKI remains difficult to predict.^3^^,^^4^

Machine learning (ML) is growing as a promising avenue for prognosticating difficult-to-predict outcomes by leveraging the wealth of data available in electronic medical records. Earlier work applying machine learning to predict postoperative AKI has focused on using classical tabular models with cross-sectional inputs, favoring incorporation of static preoperative data and some intraoperative data, limited to information without a time component.^5–7^ Recently, there has been an increase in interest in incorporating time-sensitive data into ML models using statistical feature engineering (ie, extracting mean, minimum, etc) to integrate intraoperative inputs into classical tabular machine learning methods.^8^ The results have shown statistically significantly improved results, but with unclear clinical value.^5^^,^^9^

However, the feature engineering methods used in many of these studies strip time information from each measurement, removing structures, patterns, and metadata which may be crucial. Deep learning algorithms have improved the ability to use time-series data without feature engineering. Studies using these time-aware methods have varying model architectures, including those based on Long Short-Term Memory (LSTM), recurrent neural networks (RNN), and multipath convolutional neural networks (MPCNN).^10–13^ Promisingly, these time-aware methods have outperformed conventional machine learning techniques in the prediction of postoperative length of stay.^10^

## Objectives

To date, there has yet to be a study on the efficacy of a predictive model that combines both tabular preoperative data with time-series intraoperative data to predict AKI in a general noncardiac population with a large sample size. Our study aims to (1) evaluate whether inclusion of time-series intraoperative data improves the discriminatory performance of ML models predicting postoperative AKI compared to preoperative data alone and (2) compare the performance of time-aware LSTM models and various classical feature engineering (time-invariant) ML models in predicting postoperative AKI.

## Materials and methods

### Setting and data sources

This study was conducted using the publicly available INSPIRE dataset, derived from the VitalDB dataset, which contains data from half the patients undergoing surgery at Seoul National University Hospital from 2011 to 2020.^14^^,^^15^ The dataset is available from PhysioNet, and all authors with direct access to the data signed the Data Use Agreement.^16^^,^^17^ This study complies with the 2024 TRIPOD+AI reporting requirements.^18^ This study was performed under the license administered by the owners of the dataset and falls under the Common Rule exemption for IRB due to the use of de-identified publicly accessible data. All analyses were performed in Python 3.10 and supported by NSF ACCESS.^19^^,^^20^ Patients were not involved in the design of this study. The study was not pre-registered.

### Study cohort

**Figure 1.**
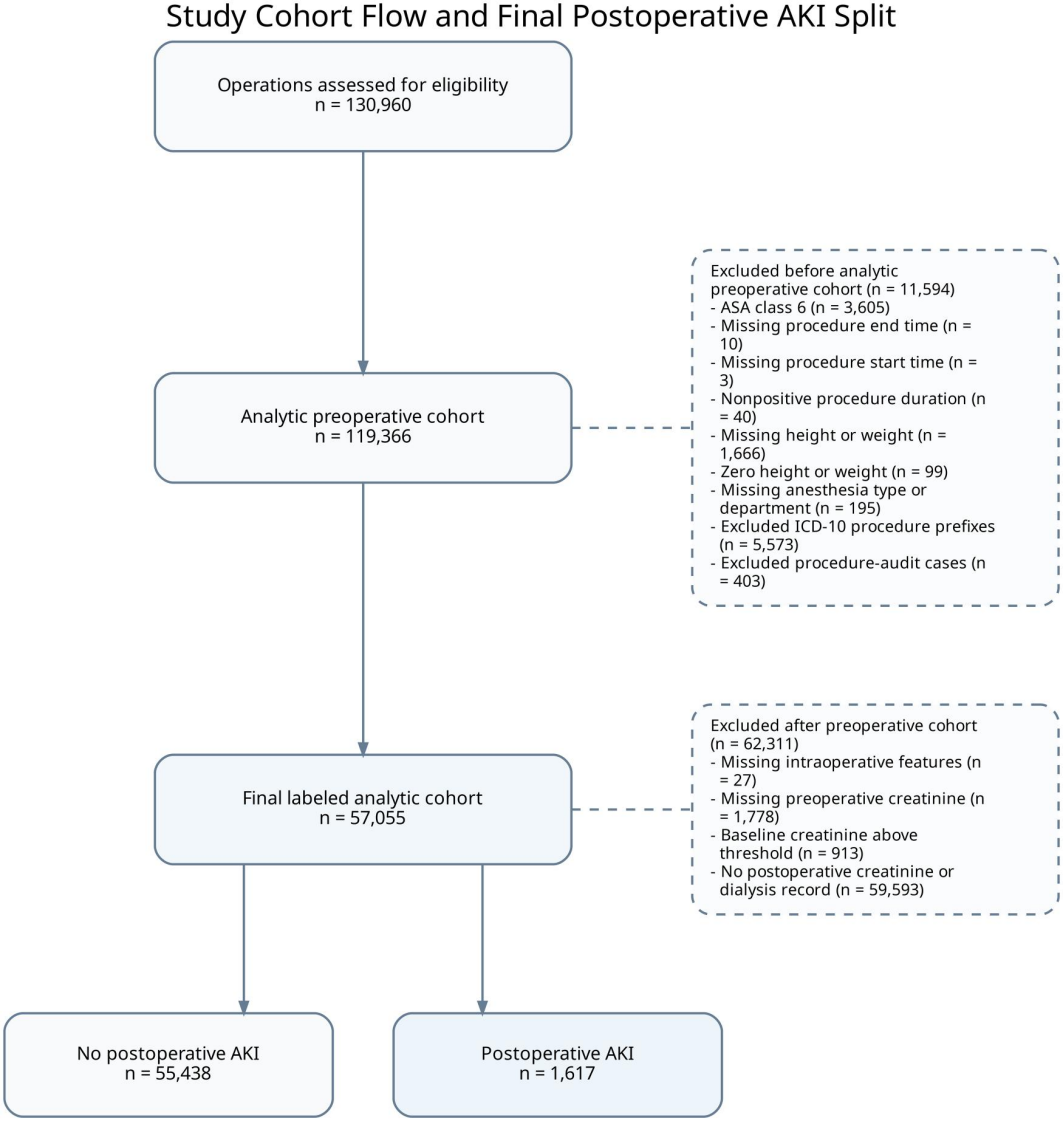
Cohort selection flowchart.

This study included all adult (>18 years) patients undergoing surgery with at least one serum creatinine (sCr) within 90 days before the day of surgery and at least one sCr within 48 hours following the operation. Obstetric, kidney donor/recipient, arteriovenous fistula operations, local anesthesia, and definite cardiac/great-vessel operations were excluded. Any operations without operation start/end times, patients with ASA-PS status of 6 (deceased organ transplant), unrealistic height or weight as recorded as zero, or patients with pre‑existing end‑stage renal disease (defined as preoperative sCr > 4.5 mg/dl corresponding to an eGFR of ≤ 15) were also excluded. “Cardio-Thoracic Surgery” was treated as an administrative department label rather than a definitive cardiac-procedure indicator; cardio-thoracic-labeled operations were audited at the procedure-code level, and retained cases did not include definite cardiac/great-vessel procedures under the prespecified audit rules. Sample size was determined by the total available cases in the database, adhering to the inclusion criteria. Detailed cohort derivation criteria are provided in Supplementary File 1. Cohort selection is summarized in Figure 1.

### Outcome variable derivation

Derivation of AKI was based on KDIGO criteria. To focus on predicting clinically important AKI, we split our outcomes into negative outcome (No AKI or Stage 1 AKI) versus positive outcome (Stage 2 AKI or Stage 3 AKI), since morbidity and mortality are significantly increased between Stage 1 and Stage 2 AKI.^21^^,^^22^ We utilized the nearest preoperative sCr as our baseline and compared it with the highest postoperative sCr within 48 hours or 7 days after the end of surgery based on KDIGO criteria. We categorized patients who received postoperative dialysis as Stage 3 AKI. Detailed AKI derivation criteria can be found in Supplementary File 2.

### Missing data

To limit sparsely collected preoperative predictors, we only selected preoperative variables from INSPIRE with <30% missing rate, which is criteria that has been used in previous studies.^23^ All intraoperative variables were included regardless of missing rate. Preoperative and intraoperative variables with more than 10% missing values were imputed to value of -99, effectively acting like dummy indicator imputation, while not adding excessive dimensionality, while missing preoperative variables with less than 10% missing values were imputed using K-Nearest Neighbor imputation (KNN). This method has been previously validated in the literature.^24^ Because sentinel encoding can allow models to learn missingness patterns, we performed a sensitivity analysis for the combined GBT model using median imputation plus explicit missingness indicators for high-missingness variables (Supplementary File 3). Other detailed input variable handling information is available in Supplementary File 4.

### Outlier detection

Outlier detection was performed prior to data processing and feature engineering using a method previously validated in the literature.^25^ Outliers were identified and adjusted by replacing the highest 1% with random values from the 95th to 99.5th percentile range and the lowest 1% with random values from the 0.5th to 5th percentile range.

### Data processing

Preoperative data included preoperative labs, patient demographics, anesthesia type, and other data collected that remained fixed during the surgery. Intraoperative data included physiologic monitoring (blood pressure, oxygen saturation, respiratory rate, etc.) collected at 5- or 10-minute intervals and transfused products. Preoperative models used variables available before surgery; intraoperative and combined models generated end-of-case predictions using intraoperative information available through completion of surgery. A description of data types and availability rates can be found in Supplementary File 5. The cohort was split into 80:20 training and testing sets.

We used the recorded ASA-PS and an adapted General Surgery Acute Kidney Injury (GS-AK) risk index as clinical baseline comparators.^26^ We used a threshold of an ASA score of ≥ 3 to predict postoperative AKI. This threshold has previously been shown to have a significant association with the development of postoperative AKI in noncardiac surgeries.^27^^,^^28^ The adapted GS-AKI score was implemented deterministically and was not refit or recalibrated in INSPIRE. It used nine grouped factors available before operation start: age ≥56 years, male sex, emergency surgery, intraperitoneal surgery proxy, diabetes, congestive heart failure, ascites, hypertension, and preoperative creatinine ≥1.2 mg/dL. Diagnosis-derived factors used only records charted before operation start while congestive heart failure and ascites were limited to a 30-day preoperative window. The GS-AKI count was used as the ordinal prediction score for discrimination, with Class III or higher (count ≥4) treated as the prespecified high-risk threshold. Detailed adaptation rules and incidence by score/class are provided in Supplementary File 6.

### Feature engineering

Feature engineering was based on previously published work.^9^ Non-binary categorical variables were encoded using one-hot encoding. Continuous variables were normalized by reporting their z-score. Due to the variety of intraoperative data, different strategies were used. Eight statistical features were computed for data reported at regular frequencies (every five or 10 minutes): minimum, maximum, mean, entropy, energy, kurtosis, skewness, and trend and then normalized. For data reported sparsely, such as ventilator machine settings or product infusion events, either the mean or sum was calculated for the whole operation, depending on clinical significance of the variable. For example, summation of total blood product transfusion is appropriate, while average of bispectral index is similarly relevant. The time-variant model ingests a variable-length multivariate sequence (T × P) of intraoperative measurements sampled every 5-10 minutes, where *T* scales with case duration, whereas the preoperative cross-sectional model uses a single fixed row per case that is independent of surgery length.

For our time-variant model, only time-series variables recorded at a regular (five or 10 minutes) frequency were passed to the LSTM, while the MLP received any single input variables, including the mean or sum of intraoperative events. Specific variables used in creation of models and data handling are described in Supplementary File 7.

### Data imbalance handling

To account for the low positive outcome rate in our population, we modified the pertinent class weights during model training to overweight the importance of our positive outcomes, as is recommended by current machine learning literature.^29^ Our minority: majority class was fixed at 1:1.

### Model creation

#### Classical time-invariant machine learning

Time-invariant ML models representing the most popular model types in the literature were tested. Logistic Regression with Ridge Regularization [LR], SVM Ensemble [SVM], Random Forests [RF], K-Nearest Neighbors [KNN], and Multilayer Perceptron [MLP] were implemented using the scikit-learn library.^30^ Different regularization methods were tested for Logistic Regression, including no regularization, L1 LASSO, L2 Ridge, and ElasticNet, and L2 Ridge was ultimately selected due to the methods’ similar performance and Ridge regularization’s improved training times (Supplementary File 8). Gradient Boosted Tree [GBT] method was implemented using the XGBoost library.^31^ All models had Bayesian hyperparameter optimization (HPO) with Tree-structured Parzen Estimator using Optuna with the balanced accuracy evaluation metric.

#### AutoML ensemble model

To represent contemporary high-performing tabular methods, an ensemble model was created using the AutoGluon^32^ library with parameters in line with the rest of the tabular models, with balanced accuracy as the evaluation metric, best quality preset, and 10-minute training time.

#### Time-variant machine learning

We implemented contemporary time-variant deep learning methods to natively interpret the time-series intraoperative data: Recurrent Neural Network (RNN), LSTM [with varying architectures, which included a Bidirectional LSTM (BiLSTM), stacked BiLSTMs, single and multiheaded attention, and Squeeze-and-Excitation (SE) Block], Transformer, and Temporal Convolutional Network (TCN) using intraoperative data only (Supplementary File 9-12). Given the similar performance between the model types, we utilized an LSTM for integration into a hybrid model. A time-aware model fused an LSTM (handling intraoperative sequences) with an MLP (handling preoperative data), with outputs fed into a final MLP with architectures optimized by Optuna. This model was implemented using the PyTorch library.^33^ Model architecture is described in Supplementary File 13.

### Model performance and evaluation

All model evaluation used stratified patient-grouped nested 5-fold cross-validation. In each outer fold, all operations from the same patient were assigned exclusively to either training or test data, preventing patient-level leakage across folds. Hyperparameter tuning and post-hoc isotonic calibration were performed within the training data using grouped inner folds. A model-specific probability threshold was selected to maximize the F2-score in training data and then applied to held-out outer-fold predictions. We report AUROC, AUPRC, sensitivity, specificity, precision, F1-score, balanced accuracy, and calibration curves.

### SHapley additive exPlanations for model interpretation

To provide a clinically interpretable method for understanding model features, we utilized SHapley Additive exPlanations (SHAP) values to determine the contribution of specific variables to model outputs using a GBT with combined preoperative and intraoperative features. SHAP values were also calculated for RF, SVM, and LR, with beeswarm plots reported in Supplementary File 14.

### Comparing model performance values

To formally compare model AUROC, we used DeLong tests with a significance level set at *P* < .05 to compare models trained on preoperative data alone with models trained with combined preoperative and intraoperative data (Supplementary File 15). Because very small AUROC differences may be statistically significant in large samples, we interpreted model comparisons primarily by effect size and confidence intervals. Decision Curve Analysis (DCA) was performed on the AutoGluon model to examine the net reclassification benefit of switching model inputs (Supplementary File 16).

## Results

### Demographics

In the patient-grouped evaluation cohort, 46 204 patients underwent 57 055 eligible operations, of which 1617 operations (2.83%) and 1124 patients (2.43%) developed stage 2/3 postoperative AKI (Table 1).

**Table 1.** Characteristics of cohort.

Characteristic	Finding
Total patients, *n*	46 204
Total operations, *n*	57 055
Age, y, mean (SD)	58.06 ± 15.16
Weight, kg, mean (SD)	63.00 ± 11.74
Height, cm, mean (SD)	162.31 ± 9.00
BMI, kg/m^2^, mean (SD)	23.88 ± 3.65
BSA, m^2^, mean (SD)	1.68 ± 0.19
ASA, mean (SD)	1.81 ± 0.62
Number of preexisting cardiac diagnoses, mean (SD)*	0.94 ± 4.96
Booking case length, min, mean (SD)	212.62 ± 110.13
Female sex, *n* (%)	22 099 (47.83%)
ASA classification, *n* (%)	
1	13 741 (29.74%)
2	27 848 (60.27%)
3	4338 (9.39%)
4	258 (0.56%)
5	19 (0.04%)
Patients with postoperative AKI, *n* (%)	1124 (2.43%)
Operations with postoperative AKI, *n* (%)	1617 (2.83%)
Department, *n* (%)	
Anesthesiology	2 (0.00%)
Cardio-Thoracic Surgery	4596 (9.95%)
Dermatology	1 (0.00%)
Emergency Medicine	1 (0.00%)
General Surgery	14 071 (30.45%)
Internal Medicine	23 (0.05%)
Neurosurgery	7303 (15.81%)
Obstetrics & Gynecology	1374 (2.97%)
Oto-laryngology	1378 (2.98%)
Orthopedic Surgery	10 929 (23.65%)
Ophthalmology	366 (0.79%)
Plastic Surgery	881 (1.91%)
Radiology	295 (0.64%)
Radiation Oncology	3 (0.01%)
Urology	4981 (10.78%)

a Number of ICD-10 code diagnosis with “I” prefix before the operation start time.

### Model performance

**Figure 2.**
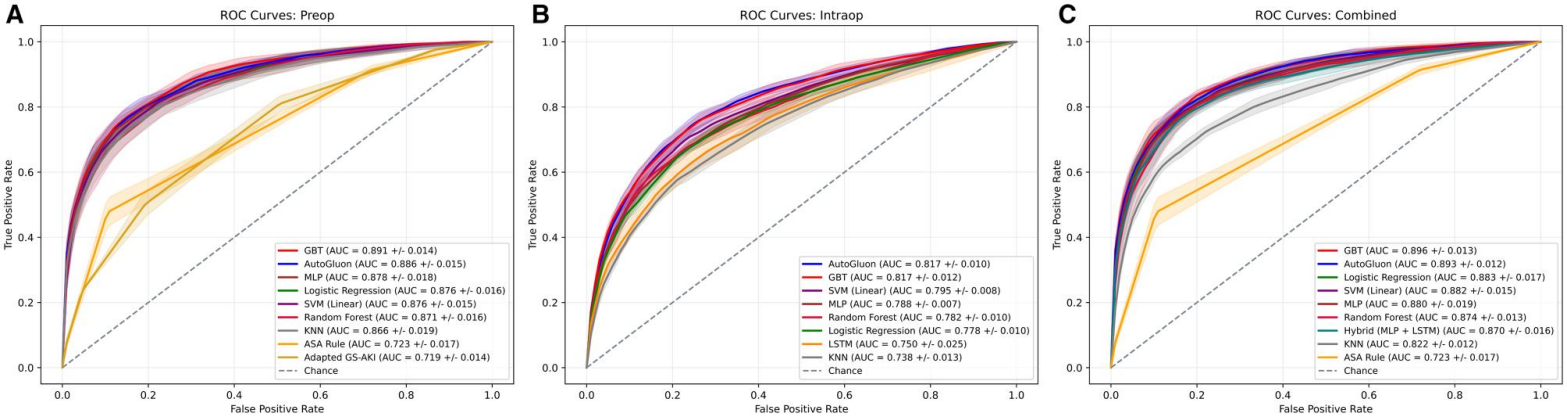
AUROC curves. Abbreviations: ASA, American Society of Anesthesiology Physical Status Score Threshold ≥ 3; GBT, Gradient Boosting Tree; LR, Logistic Regression w/Ridge; RF, Random Forest; MLP, Multilayer Perceptron; SVM, Support Vector Machine; KNN, K-Nearest Neighbors; MLP+LSTM, MLP (Preoperative) + Long Short-Term Memory (Time-Series Intraoperative) Hybrid Model.

All machine-learning models outperformed ASA-PS ≥3 and adapted GS-AKI clinical baselines. The best-performing model was combined-data GBT (AUROC 0.896, 95% CI, 0.878-0.914), followed by combined AutoGluon (0.893, 95% CI, 0.877-0.909) and preoperative-only GBT (0.891, 95% CI, 0.871-0.910). ASA-PS ≥3 (AUROC 0.723, 95% CI, 0.700-0.746) and adapted GS-AKI (AUROC 0.719, 95% CI, 0.700-0.739) underperformed machine-learning models. Intraoperative-only models had lower discrimination, with a best AUROC of 0.817. The hybrid MLP+LSTM model did not outperform simpler tabular models (AUROC 0.870, 95% CI, 0.848-0.892). Calibration curves showed relatively stable calibration for preoperative and combined models, whereas intraoperative-only models were less stable at higher predicted probabilities, particularly for LSTM, random forest, and logistic regression. We report detailed performance metrics in Table 2, AUROC curves in Figure 2, and AUPRC and calibration curves in Supplementary File 17.

**Table 2.** Detailed model performance metrics.

Model	AUROC	AUPRC	Sensitivity	Specificity	Precision	F-score	Accuracy
ASA Rule	0.723 (0.700, 0.746)	0.126 (0.092, 0.160)	0.477 (0.416, 0.537)	0.895 (0.893, 0.898)	0.117 (0.098, 0.137)	0.188 (0.159, 0.218)	0.686 (0.656, 0.716)
GS-AKI	0.719 (0.700, 0.739)	0.098 (0.085, 0.112)	–	–	–	–	–
Preoperative data							
AutoGluon	0.886 (0.865, 0.907)	0.377 (0.327, 0.428)	0.571 (0.531, 0.610)	0.948 (0.944, 0.953)	0.245 (0.205, 0.286)	0.343 (0.296, 0.389)	0.760 (0.738, 0.781)
GBT	0.891 (0.871, 0.910)	0.373 (0.313, 0.432)	0.554 (0.505, 0.602)	0.955 (0.941, 0.969)	0.272 (0.207, 0.338)	0.363 (0.303, 0.422)	0.755 (0.732, 0.777)
KNN	0.866 (0.839, 0.892)	0.341 (0.271, 0.411)	0.557 (0.500, 0.614)	0.945 (0.940, 0.949)	0.228 (0.194, 0.262)	0.323 (0.281, 0.366)	0.751 (0.723, 0.779)
Logistic Regression	0.876 (0.854, 0.898)	0.301 (0.251, 0.350)	0.595 (0.526, 0.664)	0.938 (0.925, 0.951)	0.221 (0.184, 0.258)	0.321 (0.280, 0.362)	0.767 (0.736, 0.797)
MLP	0.878 (0.852, 0.903)	0.364 (0.288, 0.441)	0.567 (0.508, 0.625)	0.951 (0.943, 0.958)	0.253 (0.210, 0.295)	0.348 (0.301, 0.396)	0.759 (0.730, 0.787)
Random Forest	0.871 (0.848, 0.894)	0.322 (0.252, 0.392)	0.540 (0.449, 0.631)	0.949 (0.936, 0.961)	0.237 (0.198, 0.276)	0.328 (0.280, 0.376)	0.744 (0.702, 0.787)
SVM (Linear)	0.876 (0.855, 0.897)	0.298 (0.245, 0.351)	0.576 (0.537, 0.614)	0.944 (0.940, 0.948)	0.231 (0.200, 0.262)	0.330 (0.293, 0.367)	0.760 (0.739, 0.781)
Intraoperative data							
AutoGluon	0.817 (0.804, 0.831)	0.195 (0.162, 0.228)	0.495 (0.424, 0.566)	0.915 (0.896, 0.934)	0.147 (0.123, 0.170)	0.225 (0.196, 0.254)	0.705 (0.676, 0.733)
GBT	0.817 (0.800, 0.833)	0.205 (0.175, 0.235)	0.495 (0.475, 0.516)	0.921 (0.915, 0.927)	0.154 (0.136, 0.172)	0.235 (0.212, 0.259)	0.708 (0.696, 0.720)
KNN	0.738 (0.720, 0.756)	0.121 (0.108, 0.135)	0.409 (0.399, 0.420)	0.897 (0.889, 0.905)	0.104 (0.097, 0.110)	0.166 (0.158, 0.173)	0.653 (0.649, 0.657)
LSTM	0.750 (0.714, 0.785)	0.153 (0.128, 0.178)	0.402 (0.287, 0.517)	0.908 (0.858, 0.957)	0.116 (0.094, 0.137)	0.176 (0.157, 0.195)	0.655 (0.619, 0.691)
Logistic Regression	0.778 (0.764, 0.792)	0.158 (0.131, 0.184)	0.443 (0.398, 0.489)	0.919 (0.908, 0.929)	0.138 (0.127, 0.148)	0.210 (0.193, 0.226)	0.681 (0.662, 0.700)
MLP	0.788 (0.778, 0.798)	0.180 (0.140, 0.219)	0.463 (0.397, 0.529)	0.918 (0.903, 0.932)	0.141 (0.128, 0.155)	0.216 (0.195, 0.237)	0.690 (0.663, 0.717)
Random Forest	0.782 (0.767, 0.796)	0.155 (0.136, 0.174)	0.474 (0.433, 0.516)	0.910 (0.897, 0.924)	0.135 (0.108, 0.163)	0.210 (0.175, 0.244)	0.692 (0.672, 0.712)
SVM (Linear)	0.795 (0.783, 0.807)	0.182 (0.153, 0.212)	0.437 (0.416, 0.458)	0.927 (0.918, 0.935)	0.149 (0.130, 0.168)	0.222 (0.199, 0.244)	0.682 (0.672, 0.692)
Combined data							
AutoGluon	0.893 (0.877, 0.909)	0.395 (0.352, 0.439)	0.593 (0.535, 0.651)	0.949 (0.943, 0.955)	0.253 (0.235, 0.272)	0.355 (0.328, 0.382)	0.771 (0.744, 0.798)
GBT	0.896 (0.878, 0.914)	0.390 (0.334, 0.447)	0.585 (0.534, 0.636)	0.951 (0.940, 0.962)	0.264 (0.209, 0.319)	0.362 (0.305, 0.418)	0.768 (0.743, 0.794)
Hybrid (MLP + LSTM)	0.822 (0.806, 0.838)	0.214 (0.180, 0.248)	0.542 (0.513, 0.571)	0.919 (0.905, 0.933)	0.166 (0.136, 0.196)	0.253 (0.218, 0.287)	0.731 (0.718, 0.743)
KNN	0.883 (0.859, 0.906)	0.307 (0.253, 0.360)	0.607 (0.561, 0.654)	0.942 (0.934, 0.950)	0.235 (0.194, 0.275)	0.338 (0.290, 0.386)	0.774 (0.750, 0.799)
Logistic Regression	0.880 (0.853, 0.906)	0.334 (0.263, 0.404)	0.574 (0.547, 0.602)	0.947 (0.934, 0.960)	0.247 (0.175, 0.319)	0.343 (0.272, 0.413)	0.761 (0.743, 0.779)
MLP	0.870 (0.848, 0.892)	0.322 (0.251, 0.393)	0.539 (0.451, 0.628)	0.952 (0.940, 0.963)	0.241 (0.202, 0.279)	0.331 (0.287, 0.376)	0.745 (0.704, 0.787)
Random Forest	0.874 (0.856, 0.892)	0.316 (0.249, 0.383)	0.574 (0.506, 0.641)	0.940 (0.930, 0.950)	0.221 (0.163, 0.279)	0.318 (0.248, 0.389)	0.757 (0.719, 0.794)
SVM (Linear)	0.882 (0.860, 0.903)	0.305 (0.250, 0.359)	0.575 (0.504, 0.646)	0.948 (0.939, 0.957)	0.245 (0.212, 0.278)	0.343 (0.302, 0.383)	0.761 (0.728, 0.795)

Mean (95% CI); Abbreviations: ASA, American Society of Anesthesiology Physical Status Score Threshold ≥ 3; GS-AKI, General Surgery Acute Kidney Injury Risk Index; GBT, Gradient Boosting Tree; LR, Logistic Regression w/Ridge, RF, Random Forest; MLP, Multilayer Perceptron; SVM, Support Vector Machine; KNN, K-Nearest Neighbors; MLP+LSTM, MLP (Preoperative) + Long Short-Term Memory (Time-Series Intraoperative) Hybrid Model.

#### Reclassification analysis

Supplementary File 16 examines the number of patients misclassified by each model type (ie, the number of patients who were AKI positive but predicted to be AKI negative). We found that 35-45% of patients were reclassified correctly when moving from training on intraoperative data only to preoperative data but moving from preoperative data to combined data led to a net reclassification of 9-23%, a much more modest improvement.

#### Decision curve analysis

Supplementary File 17 shows the decision curve analysis of varying inputs on the AutoGluon GBT models. DCA showed nearly overlapping net-benefit curves for the Combined and Preoperative models across clinically relevant thresholds (≈3-15%), indicating no material clinical advantage of adding intraoperative variables, while the intraoperative-only model was inferior to both.

### Model interpretation

The SHAP values can be interpreted to provide clinical insights as to how the model predicts postoperative AKI. We provide variable thresholds (Figure 4) where postoperative AKI risk is higher.

#### Variable thresholds

To investigate the effect of continuous variables on the prediction of postoperative AKI across the entire dataset, we plotted the SHAP contribution of each variable against the true value of the continuous variable (Figure 4). We plotted linear trend lines based on both negative and positive cases to identify thresholds that were associated with positive SHAP values contributing to a higher risk of postoperative AKI.

## Discussion

The goal of this study was to determine the best model architecture and input data to predict postoperative AKI. We compared preoperative information against intraoperative information and assessed the benefits and drawbacks of combining them. Two approaches to intraoperative data were tested: feature engineering into summary variables and direct input into modern time-sensitive machine learning models. To our knowledge, this is the first study to compare these approaches at this scale for AKI prediction.

When using only preoperative tabular data, tree-based models and AutoGluon consistently performed best, corroborating prior work that these methods are well-suited for structured clinical data. Logistic Regression also performed competitively, underscoring that interpretable models may remain viable baselines aligning with previous work.^34^ AutoGluon’s ease of use and bagged ensemble approach are attractive features for routine use. Tree-based models and SHAP calculations make them desirable for easily interpretable feature importance, similar to logistic regression coefficient extraction. Clinically, these findings suggest that strong predictive performance is achievable preoperatively.^35^

Intraoperative data, when transformed into summary features, performed worse than preoperative variables alone, but modestly improved discrimination when combined. For most models, adding intraoperative information provided mostly nonsignificant gains in discrimination and model performance suggesting some incremental value but limited clinical impact at the current data resolution, in line with the current literature.^5^^,^^9^^,^^25^^,^^36^^,^^37^ As the intraoperative data we evaluated was aggregated at the end of surgery, predictions of these models could be used to inform postoperative management such as decisions about monitoring intensity, medication review, nephrotoxin avoidance, fluid management, and early nephrology involvement for very high-risk patients.

Our study is among the first to directly compare modern time-aware deep learning techniques, such as RNN, LSTM, Transformer, and TCN, on intraoperative data. Given our results (Supplementary File 9), we chose to fuze LSTM with MLP, testing combined preoperative and time-aware intraoperative data because of the performance-to-computational efficiency ratio of LSTM and the native ability to pass output vectors from LSTM and MLP to a final decision layer, allowing all models to be trained concurrently. Despite their theoretical strengths, these architectures underperformed and had substantially higher computational costs. However, this comparison was not compute-matched: the deep-learning models received a substantially larger tuning and training budget than AutoGluon, so these results should be interpreted as performance under our implemented training framework rather than as a resource-equivalent benchmark. Advanced implementations, such as bidirectional LSTMs, single- or multi-head attention, and squeeze-and-excitation blocks, showed no meaningful performance improvement. Such architectures typically excel on high-frequency waveforms (≥125 Hz), and we suspect the temporal resolution of most intraoperative data (<1 Hz) prevents these models from extracting useful physiologic information, amplifying noise instead.

**Figure 3.**
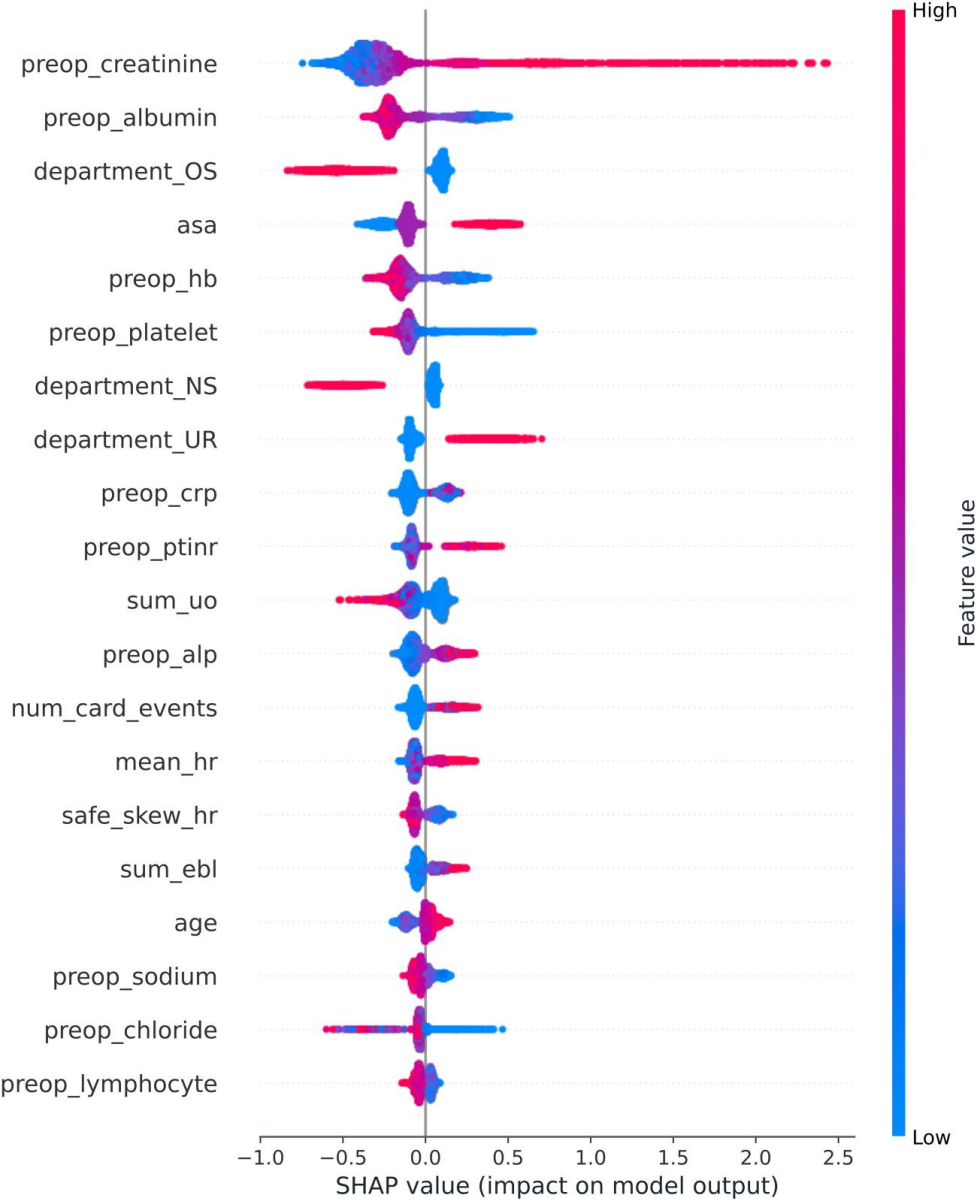
SHAP Interpretation of GBT trained on combined pre- and intraoperative data.

**Figure 4.**
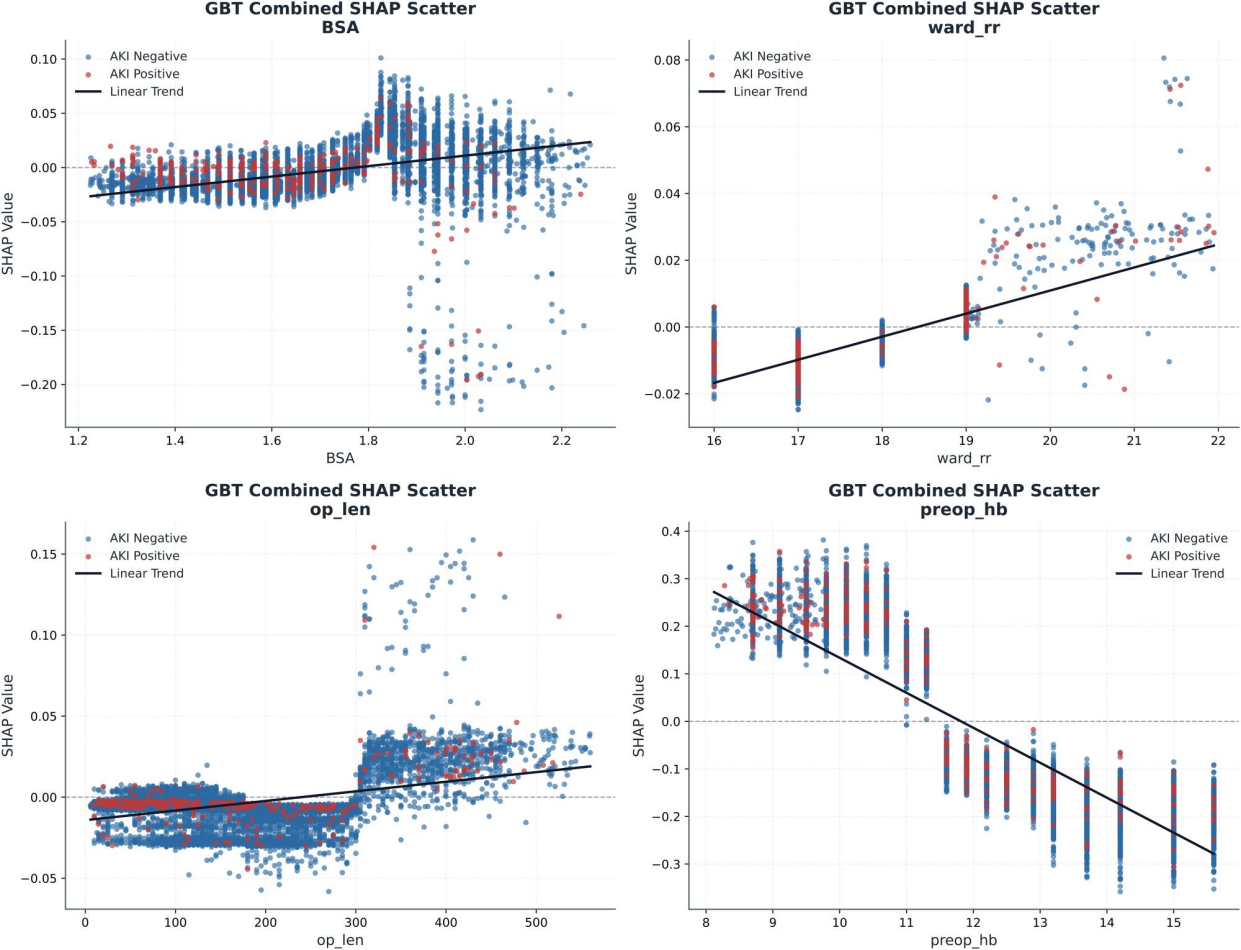
Threshold for laboratory values and physiological measurements. The true value of the measurement is plotted on the X-axis, while the SHAP value is plotted on the Y-axis. Positive SHAP values represent positive contributions to the likelihood of AKI, while negative SHAP values represent negative contributions. Blue points represent true negative AKI cases and red points represent true positive AKI cases, with each point representing a patient case from the test cohort. Abbreviations: BSA, Body surface area; op_len, operation length; preop_hb, preoperative hemoglobin; ward_rr, nearest recorded respiratory rate from operation start.

Interpretability remains crucial for clinical adoption. Using SHAP analysis and regression coefficients, we identified consistent predictors of AKI across model types. Figure 4 highlights several such predictors, including preoperative factors (eg, hemoglobin ∼< 11.9 g/dL, respiratory rate >∼ 18.3 respirations/min, BSA >∼ 1.8) and intraoperative events (eg, operation length >∼ 220 min). The waterfall plot (Figure 3) provides clinical validity, showing the model ranks clinically important features as important to prediction (preoperative creatinine and albumin, hemoglobin, and platelet, CRP and intraoperative variables such as total urine output). Importantly, all models outperformed clinical baselines such as ASA classification or GS-AKI, suggesting that machine learning approaches may complement physician judgment and preexisting risk stratification scales by integrating a broader set of risk factors. Clinically, modifiable factors were found in both the preoperative and intraoperative phases. Preoperatively, anemia is commonly addressed through hematology clinics with iron therapy, weight loss is always encouraged, especially for bariatric surgery, and adequate nutritional status is always preferred. The intraoperative factors that emerged may be managed. For example, fluid status and vasopressor use may augment blood pressure to maintain perfusion, electrolytes may be administered to keep patients within normal ranges, inspired oxygen fraction may be adjusted to normalize values, etc. However, maintaining normal ranges may not be the answer to these issues. For example, the use of furosemide to maintain intraoperative urine output has been shown to increase AKI rates, suggesting that additional interventions to normalize physiological variables may confer harm.^38^ Of course, for each modifiable intraoperative variable with suggested impact, additional studies may need to be performed to investigate the impact of pharmacological therapy.

Future work should focus on integrating higher-resolution intraoperative data, such as raw physiologic waveforms from repositories like VitalDB, which may enable time-aware models to extract meaningful temporal patterns rather than noise. Advanced feature engineering libraries (such as catch22) and other transfer learning approaches could further enhance the value of intraoperative information. A logical next step is to define the minimum sampling rate required to confer clinically meaningful gains, which would guide perioperative data collection standards. Despite these opportunities, our study has important limitations. The data were derived from a single South Korean academic center, limiting geographic, demographic, practice-pattern, and health-system generalizability. Fairness was not assessed, and the relatively low incidence of AKI constrained the sample size despite the use of imbalance corrections. SHAP analysis should be interpreted cautiously, especially department indicators, which should be interpreted as administrative case-mix proxies rather than causal surgical mechanisms. The missingness sensitivity analysis showed minimal change in discrimination when high-missingness variables were handled with median imputation and explicit missingness indicators but features with substantial missingness may still reflect documentation and monitoring patterns as well as physiology. Calibration was more stable for preoperative and combined models than for intraoperative-only models, supporting caution when using intraoperative-only predictions for probability-based clinical decisions. While the use of clinically stage 2/3 postoperative AKI emphasized events more likely to affect postoperative management, it limits direct comparison with literature predicting any-stage AKI. Additionally, heterogeneity in data reporting prevented external validation against independent cohorts. Addressing these limitations through multi-institutional collaborations with larger and higher-resolution datasets will be essential to determine whether more complex modeling approaches can meaningfully improve perioperative AKI prediction and support clinical decision making.

## Conclusion

Taken together, our results indicate that preoperative variables remain the strongest predictors of postoperative AKI, and intraoperative data with its current temporal resolution confers only modest incremental value. Regarding model selection, the most practical choices were AutoGluon for ease of optimization and tree-based models for increased interpretability. Modern time-aware deep learning models do not outperform simpler methods at existing data resolutions. Clinically, this supports focusing on preoperative risk stratification and optimization while recognizing the potential for future gains if higher-resolution intraoperative data can be leveraged along with future external cohort validation.

## Supplementary Material

ooag092_Supplementary_Data

## Data and code availability statement

The third-party source data underlying this article are available from the INSPIRE database on PhysioNet at https://doi.org/10.13026/46m4-f655, subject to PhysioNet credentialing and data use requirements. Derived data and reproducibility materials supporting the analyses have been deposited in the Dryad Digital Repository at https://doi.org/10.5061/dryad.z08kprrw7. Analysis code has been archived in Zenodo at https://doi.org/10.5281/zenodo.20357628.

## Presentations

This work has been previously presented as an oral presentation at ASA 2025.
