# Supplementary material for: Integration of intraoperative data in interpretable machine learning models to predict postoperative AKI in noncardiac surgery patients
Source: JAMIA Open. 2026 Jun 20;9(3):ooag092. doi: 10.1093/jamiaopen/ooag092 (PMC13282712; doi:10.1093/jamiaopen/ooag092)

**Integration of Intraoperative Data in Interpretable Machine Learning Models to Predict Postoperative AKI in Noncardiac Surgery Patients Supplemental File**

## Supplemental File 1: Detailed Derivation of Study Cohort Inclusion/Exclusion Criteria

Inclusion criteria included adult operative cases during the study period with ≥1 serum creatinine (sCr) value in the 90 days preceding surgery and ≥1 serum sCr in the 48 h following surgery

- labs.csv
  - creatinine
    - <90 days before surgery, <48 hours after surgery
      - Remember surgery is defined by opstart_time and opend_time in operations.csv
  - >=18 year olds
    - Age, operations.csv
- operations.csv
  - antype = General
    - Neuraxial, MAC, Local
      - Exclude Local
      - General vs Not General as a binary variable

Exclusion criteria included obstetric, kidney donor and recipient, and arteriovenous fistula cases due to preexisting alteration in renal physiology.

- Exclude Operations without a opstart_time or a opend_time
- Exclude ASA VI
  - Deceased organ donors
- Exclude using: “icd10_pcs” variable in operations.csv
  - Obstetric
    - Any PCS code starting with “10”
  - Kidney Donor/Recipient
    - Any PCS code starting with “0TY”
  - AV Fistula
    - Any PCS code starting with “B50” or “B51”
- Exclude rows if height and weight are 0
- Last preoperative sCr > 4.5 mg/dL
  - creatinine, labs.csv, nearest to opstart_time (operations.csv)

##

### Cardio-Thoracic Surgery Department-Label Procedure-Code Audit

Because the hospital department field is an administrative service label rather than a definitive operative phenotype, cardiothoracic-labeled cases were audited at the operation level. The audit used the final analytic operation identifiers (`op_id`) and joined them back to `operations.csv` to evaluate the 5-character `icd10_pcs` procedure prefix, department label, and cardiopulmonary bypass (CPB) timing fields (`cpbon_time` and `cpboff_time`). The procedure prefixes were interpreted using a CMS ICD-10-PCS order-file reference collapsed to the observed 5-character prefix level.

Definite cardiac procedures were identified by official Heart and Great Vessels procedure families, including `02...` ICD-10-PCS prefixes, or by explicit cardiac procedure-title matches. Clearly noncardiac thoracic procedures, including respiratory-system (`0B...`) and other thoracic, mediastinal, foregut, or chest-related noncardiac families without CPB discordance, were retained. Vascular families without CPB were retained but described separately. Residual cases with CPB support, unresolved prefixes, malformed or missing procedure prefixes, or discordant procedure/CPB patterns were routed to structured gray-zone categories and conservatively excluded unless they were CPB-negative and supported by a benign same-4-character neighboring procedure family.

The final cohort rule therefore did not exclude cardiothoracic-labeled cases solely because of department assignment. Instead, it excluded definite cardiac procedures and residual CPB-supported or unresolved ambiguous cases, while retaining clearly noncardiac thoracic and other clearly noncardiac cardiothoracic-service operations.

| **Audit category** | **Operational definition** | **Final cohort action** |
| --- | --- | --- |
| Definite cardiac procedure | Heart and Great Vessels (02...) ICD-10-PCS prefix or explicit cardiac procedure-title family | Exclude |
| Clearly noncardiac thoracic procedure | Respiratory (0B...), thoracic, mediastinal, foregut, or chest-related noncardiac family without CPB discordance | Retain |
| Clearly noncardiac CTS-service procedure | Cardiothoracic-service case with noncardiac nonthoracic procedure family and no CPB discordance | Retain |
| Vascular family without CPB | Non-02 vascular family without CPB timing populated | Retain and describe |
| CPB-supported or unresolved gray-zone case | CPB-supported respiratory/vascular/nonvascular discordance, unresolved prefix/title, or malformed/missing procedure prefix | Exclude conservatively unless CPB-negative with benign same-4-character neighbor support |

##

## Supplemental File 2: Outcome Variable Derivation

- Acute Kidney Injury
  - Models should predict the maximum difference between preoperative creatinine and postoperative creatinine within 48 hours of the operation
    - creatinine, labs.csv, subtract the largest creatinine value in the 48 hours following opend_time (operations.csv) from the preop_creatinine that we derived previously
    - Cases with a postoperative sCr decrease were labeled as having a postoperative sCr increase of 0 for the purposes of modeling.
  - The modeled binary outcome was stage 2/3 postoperative AKI: no AKI or stage 1 AKI was coded as the negative class, and stage 2 or stage 3 AKI was coded as the positive class. Urine output was not used for outcome adjudication because it was not reliably charted in INSPIRE.

| Stage | Serum Creatinine Criteria | Urine Output Criteria |
| --- | --- | --- |
| 1 | Creatinine 1.5 to 1.9 times baseline within 7 days, OR  Creatinine increase > 0.3 mg/dL within 48 hrs | < 0.5 ml/kg/hr x 6-12 hours |
| 2 | Creatinine 2.0-2.9 times baseline | < 0.5 ml/kg/hr for > 12 hours |
| 3 | Creatinine > 3 times baseline, OR Creatinine > 4 mg/dL, OR Initiation of dialysis | < 0.3 ml/kg/hr for > 24 hours, OR anuria > 12 hours |

- To get the “times baseline” requirement divide by already existing preop creatinine. For stage 3 derivation, add preop creatinine to the delta to get total creatinine
  - Urine output was not used for outcome adjudication because it was not reliably charted in INSPIRE; postoperative dialysis, when identified, was classified as stage 3 AKI.

## Supplemental File 3: Input Variable Handling and Missingness Sensitivity

Because variables with greater than 10% missingness were handled using sentinel encoding in the primary preprocessing pipeline, we performed a targeted sensitivity analysis to evaluate whether this encoding affected tree-based model performance or SHAP feature attribution. The analysis was restricted to the combined-data GBT model. Patient-grouped evaluation folds were preserved. Within each training fold, variables with less than 10% missingness continued to be handled using the original KNN-based imputation approach, whereas variables with greater than 10% missingness were handled using median imputation with an additional binary missingness-indicator column. Missingness indicators were generated from raw missingness before imputation, retained as 0/1 variables, and excluded from scaling. All preprocessing steps, including outlier handling, imputation, scaling, and median estimation, were fit within the training fold only. No additional hyperparameter optimization was performed.

Supplemental Results: Missingness Sensitivity Analysis

Model discrimination was stable under the alternative missingness strategy. The combined GBT AUROC changed from 0.8883 with sentinel encoding to 0.8878 with median imputation plus missingness indicators. AUPRC changed from 0.4078 to 0.3997. Threshold-dependent metrics were also similar, with small decreases in sensitivity and small increases in specificity, precision, F1 score, and accuracy.

Combined GBT Performance Under Alternative Missingness Encoding

| Metric | Sentinel encoding | Median + missingness indicator | Difference |
| --- | --- | --- | --- |
| AUROC | 0.8883 | 0.8878 | -0.0005 |
| AUPRC | 0.4078 | 0.3997 | -0.0081 |
| Sensitivity | 0.5510 | 0.5380 | -0.0130 |
| Specificity | 0.9592 | 0.9646 | +0.0054 |
| Precision | 0.2825 | 0.3070 | +0.0245 |
| F1 score | 0.3735 | 0.3909 | +0.0174 |
| Accuracy | 0.9476 | 0.9525 | +0.0049 |

SHAP rankings were generally similar, although attribution for some variables with substantial missingness was attenuated. Urine output remained contributory but moved from SHAP rank 4 to rank 11, with mean absolute SHAP decreasing from 0.2682 to 0.1314. Estimated blood loss moved from rank 11 to rank 13, and preoperative CRP moved from rank 14 to rank 19. The added missingness-indicator variables were not dominant SHAP contributors; the highest-ranking missingness indicator was the urine output missingness flag at rank 33.

SHAP Comparison for Selected High-Missingness Features

| Feature | Missing rate | Sentinel rank | Median + indicator rank | Sentinel mean \|SHAP\| | Median + indicator mean \|SHAP\| | Missingness flag rank |
| --- | --- | --- | --- | --- | --- | --- |
| Urine output | 62.9% | 4 | 11 | 0.2682 | 0.1314 | 33 |
| Estimated blood loss | 48.0% | 11 | 13 | 0.1404 | 0.1256 | 223 |
| Preoperative CRP | 42.6% | 14 | 19 | 0.1189 | 0.0918 | 139 |

Highest-Ranking Missingness Indicators in the Sensitivity Model

| Missingness indicator | Original feature | SHAP rank | Mean \|SHAP\| |
| --- | --- | --- | --- |
| sum_uo_missing_flag | Urine output | 33 | 0.0555 |
| preop_fibrinogen_missing_flag | Preoperative fibrinogen | 86 | 0.0325 |
| mean_bis_missing_flag | Mean BIS | 98 | 0.0312 |
| preop_crp_missing_flag | Preoperative CRP | 139 | 0.0207 |
| equiv_MAC_totals_missing_flag | Equivalent MAC totals | 177 | 0.0158 |

Supplemental Interpretation

These findings suggest that the combined GBT model’s predictive performance was robust to the missingness-encoding strategy. However, SHAP attribution for some incompletely observed variables, particularly urine output, was sensitive to how missingness was represented. Therefore, feature-importance results for variables with substantial missingness should be interpreted as potentially reflecting both physiologic information and patterns of documentation, monitoring, or procedural acuity. The absence of dominant missingness-indicator features suggests that model performance was not primarily driven by explicit missingness flags alone.

##

## Supplemental File 4: Special Variable Handling

### ASA Class and Emergency

- **File:** operations.csv
- **Variables:** asa, emop
- **Note:**
  - asa is coded as 1/2/3/4/5.
  - emop is a binary indicator for emergency operations.

### Booking Case Length

- **File:** operations.csv
- **Variables:** orin_time, orout_time
- **Calculation:**
  orout_time - orin_time

### Operation Length

- **File:** operations.csv
- **Variables:** opend_time, opstart_time
- **Calculation:**
  opend_time - opstart_time

### Number of Past Cardiovascular Diagnoses

- **File:** diagnosis.csv
- **Variables:** icd10_cm, chart_time
- **Steps:**
  1. Define a list of ICD-10-CM codes related to cardiovascular diagnoses.
     - All ICD-10 CM codes starting with “I” are cardiovascular disease
  2. Filter diagnosis.csv for these codes.
  3. Count the number of distinct cardiovascular diagnoses per patient where chart_time is before the opdate in operations.csv.

### Body Surface Area (BSA)

- **File:** Not directly available.
- **Calculation:** Can be calculated using height and weight with a formula like the Mosteller formula: BSA = sqr(Height * Weight / 3600)
- Body Mass Index (BMI)
- **File:** Not directly available.
- **Calculation:** Can be calculated using height and weight:
  Weight (kg) / Height (m)2

### Operation Length

1. **File:** operations.csv
2. **Variables:** opend_time, opstart_time
3. **Calculation:**
   opend_time - opstart_time

### Last Preoperative sCr within 90 Days

- **File:** labs.csv
- **Variables:** item_name (for sCr), value (sCr value), chart_time
- **Label:** creatinine
- **Steps:**
  1. Find the opdate in operations.csv.
  2. Calculate the date 90 days prior to opdate.
  3. Filter labs.csv for rows where:
     - item_name is 'creatinine'.
     - chart_time is between the calculated date and opdate.
  4. Identify the sCr measurement with the chart_time closest to opdate (but still before opdate).

1. **Total fluids in:**
   - **File:** vitals.csv
   - **Variables:** item_name, value, chart_time
   - **Labels:** WILL GIVE A VALUE in mL
     1. Sum for each op_id:
        - d5w
        - d10w
        - d50w
        - ns
        - hs
        - hns
        - alb5
        - alb20
        - hes
        - Psa
   - Add all variables, then add all values together (report aggregate)
2. **Total fluids in per minute:**
   - **File:** vitals.csv
   - **Variables:** item_name, value, chart_time
   - **Labels:** Final value will bein mL/min
     1. Take total fluids in and divide by the length of the operation (opend_time - opstart_time)
3. **Infused RBCs**
   - **File:** vitals.csv
   - **Variables:** item_name
   - **Labels:** rbc
   - Add all timepoints, report aggregate
4. **Infused Platelets**
   - **File:** vitals.csv
   - **Variables:** item_name
   - **Labels:** pc,
   - Add all timepoints, report aggregate
5. **Infused FFP**
   - **File:** vitals.csv
   - **Variables:** item_name
   - **Labels:** **ffp**
   - **Add all values together, report aggregate**
6. **Infused Cryoprecipitate**
   - **File:** vitals.csv
   - **Variables:** item_name
   - **Labels:** cryo
   - **Add all values together, report aggregate**
7. **Inhalational Anesthetics: MAC Equivalents**
   - **File:** vitals.csv
   - **Variables:** item_name
   - Use equivalent MAC values. It looks like inspire only uses desflurane and sevoflurane
     1. Desflurane: **6.6%**
     2. Sevoflurane: **1.8%**
   - Take the given value (end tidal concentration of the inhalational anesthetic), divide by the MAC value to get MAC, then add them together.

## Supplemental File 5: Variables Included and Missing Rate

| **Variable** | **Fill Rate (%)** |
| --- | --- |
| BMI | **100.00%** |
| BSA | **100.00%** |
| age | **100.00%** |
| antype | **100.00%** |
| asa | **100.00%** |
| booking_case_length | **100.00%** |
| department_AN | **100.00%** |
| department_CTS | **100.00%** |
| department_DM | **100.00%** |
| department_EM | **100.00%** |
| department_GS | **100.00%** |
| department_IM | **100.00%** |
| department_NS | **100.00%** |
| department_OG | **100.00%** |
| department_OL | **100.00%** |
| department_OS | **100.00%** |
| department_OT | **100.00%** |
| department_PS | **100.00%** |
| department_RAD | **100.00%** |
| department_RO | **100.00%** |
| department_UR | **100.00%** |
| emop | **100.00%** |
| height | **100.00%** |
| num_card_events | **100.00%** |
| op_id | **100.00%** |
| op_len | **100.00%** |
| sex | **100.00%** |
| weight | **100.00%** |
| energy_spo2 | 99.99% |
| max_spo2 | 99.99% |
| mean_spo2 | 99.99% |
| min_spo2 | 99.99% |
| safe_entropy_spo2 | 99.99% |
| safe_kurtosis_spo2 | 99.99% |
| safe_skew_spo2 | 99.99% |
| safe_trend_spo2 | 99.99% |
| energy_hr | 99.97% |
| max_hr | 99.97% |
| mean_hr | 99.97% |
| min_hr | 99.97% |
| safe_entropy_hr | 99.97% |
| safe_kurtosis_hr | 99.97% |
| safe_skew_hr | 99.97% |
| safe_trend_hr | 99.97% |
| energy_nibp_mbp | 99.46% |
| max_nibp_mbp | 99.46% |
| mean_nibp_mbp | 99.46% |
| min_nibp_mbp | 99.46% |
| safe_entropy_nibp_mbp | 99.46% |
| safe_kurtosis_nibp_mbp | 99.46% |
| safe_skew_nibp_mbp | 99.46% |
| safe_trend_nibp_mbp | 99.46% |
| energy_nibp_sbp | 99.45% |
| max_nibp_sbp | 99.45% |
| mean_nibp_sbp | 99.45% |
| min_nibp_sbp | 99.45% |
| safe_entropy_nibp_sbp | 99.45% |
| safe_kurtosis_nibp_sbp | 99.45% |
| safe_skew_nibp_sbp | 99.45% |
| safe_trend_nibp_sbp | 99.45% |
| energy_nibp_dbp | 99.45% |
| max_nibp_dbp | 99.45% |
| mean_nibp_dbp | 99.45% |
| min_nibp_dbp | 99.45% |
| safe_entropy_nibp_dbp | 99.45% |
| safe_kurtosis_nibp_dbp | 99.45% |
| safe_skew_nibp_dbp | 99.45% |
| safe_trend_nibp_dbp | 99.45% |
| preop_creatinine | 98.51% |
| ward_hr | 97.80% |
| ward_nibp_sbp | 97.42% |
| ward_nibp_dbp | 97.42% |
| ward_rr | 97.27% |
| ward_bt | 97.26% |
| energy_etco2 | 94.64% |
| max_etco2 | 94.64% |
| mean_etco2 | 94.64% |
| min_etco2 | 94.64% |
| safe_entropy_etco2 | 94.64% |
| safe_kurtosis_etco2 | 94.64% |
| safe_skew_etco2 | 94.64% |
| safe_trend_etco2 | 94.64% |
| mean_o2 | 92.39% |
| fluids_agg | 92.30% |
| preop_potassium | 91.27% |
| preop_sodium | 91.27% |
| preop_hct | 91.12% |
| preop_hb | 90.72% |
| preop_chloride | 90.38% |
| preop_wbc | 90.34% |
| preop_calcium | 90.23% |
| preop_platelet | 90.18% |
| preop_albumin | 89.74% |
| preop_bun | 89.56% |
| preop_ptinr | 89.35% |
| preop_ast | 89.29% |
| preop_alt | 89.26% |
| preop_phosphorus | 89.25% |
| preop_total_protein | 89.21% |
| preop_aptt | 89.07% |
| preop_alp | 89.06% |
| energy_rr | 88.78% |
| max_rr | 88.78% |
| mean_rr | 88.78% |
| min_rr | 88.78% |
| safe_entropy_rr | 88.78% |
| safe_kurtosis_rr | 88.78% |
| safe_skew_rr | 88.78% |
| safe_trend_rr | 88.78% |
| preop_glucose | 88.02% |
| preop_total_bilirubin | 87.96% |
| preop_lymphocyte | 84.51% |
| preop_seg | 84.49% |
| energy_fio2 | 82.75% |
| max_fio2 | 82.75% |
| mean_fio2 | 82.75% |
| min_fio2 | 82.75% |
| safe_entropy_fio2 | 82.75% |
| safe_kurtosis_fio2 | 82.75% |
| safe_skew_fio2 | 82.75% |
| safe_trend_fio2 | 82.75% |
| energy_pip | 80.98% |
| max_pip | 80.98% |
| mean_pip | 80.98% |
| min_pip | 80.98% |
| safe_entropy_pip | 80.98% |
| safe_kurtosis_pip | 80.98% |
| safe_skew_pip | 80.98% |
| safe_trend_pip | 80.98% |
| energy_minvol | 78.32% |
| max_minvol | 78.32% |
| mean_minvol | 78.32% |
| min_minvol | 78.32% |
| safe_entropy_minvol | 78.32% |
| safe_kurtosis_minvol | 78.32% |
| safe_skew_minvol | 78.32% |
| safe_trend_minvol | 78.32% |
| energy_vt | 77.61% |
| max_vt | 77.61% |
| mean_vt | 77.61% |
| min_vt | 77.61% |
| safe_entropy_vt | 77.61% |
| safe_kurtosis_vt | 77.61% |
| safe_skew_vt | 77.61% |
| safe_trend_vt | 77.61% |
| energy_bt | 77.43% |
| max_bt | 77.43% |
| mean_bt | 77.43% |
| min_bt | 77.43% |
| safe_entropy_bt | 77.43% |
| safe_kurtosis_bt | 77.43% |
| safe_skew_bt | 77.43% |
| safe_trend_bt | 77.43% |
| mean_air | 72.54% |
| energy_pmean | 66.78% |
| max_pmean | 66.78% |
| mean_pmean | 66.78% |
| min_pmean | 66.78% |
| safe_entropy_pmean | 66.78% |
| safe_kurtosis_pmean | 66.78% |
| safe_skew_pmean | 66.78% |
| safe_trend_pmean | 66.78% |
| energy_peep | 62.71% |
| max_peep | 62.71% |
| mean_peep | 62.71% |
| min_peep | 62.71% |
| safe_entropy_peep | 62.71% |
| safe_kurtosis_peep | 62.71% |
| safe_skew_peep | 62.71% |
| safe_trend_peep | 62.71% |
| preop_fibrinogen | 61.45% |
| preop_crp | 57.44% |
| sum_ebl | 52.00% |
| mean_bis | 50.09% |
| equiv_MAC_totals | 45.86% |
| energy_pplat | 41.97% |
| max_pplat | 41.97% |
| mean_pplat | 41.97% |
| min_pplat | 41.97% |
| safe_entropy_pplat | 41.97% |
| safe_kurtosis_pplat | 41.97% |
| safe_skew_pplat | 41.97% |
| safe_trend_pplat | 41.97% |
| energy_etgas | 41.17% |
| max_etgas | 41.17% |
| mean_etgas | 41.17% |
| min_etgas | 41.17% |
| safe_entropy_etgas | 41.17% |
| safe_kurtosis_etgas | 41.17% |
| safe_skew_etgas | 41.17% |
| safe_trend_etgas | 41.17% |
| sum_ppf | 39.76% |
| energy_cpat | 39.49% |
| max_cpat | 39.49% |
| mean_cpat | 39.49% |
| min_cpat | 39.49% |
| safe_entropy_cpat | 39.49% |
| safe_kurtosis_cpat | 39.49% |
| safe_skew_cpat | 39.49% |
| safe_trend_cpat | 39.49% |
| ward_spo2 | 37.50% |
| sum_uo | 37.06% |
| energy_art_mbp | 36.01% |
| max_art_mbp | 36.01% |
| mean_art_mbp | 36.01% |
| min_art_mbp | 36.01% |
| safe_entropy_art_mbp | 36.01% |
| safe_kurtosis_art_mbp | 36.01% |
| safe_skew_art_mbp | 36.01% |
| safe_trend_art_mbp | 36.01% |
| energy_art_sbp | 35.72% |
| max_art_sbp | 35.72% |
| mean_art_sbp | 35.72% |
| min_art_sbp | 35.72% |
| safe_entropy_art_sbp | 35.72% |
| safe_kurtosis_art_sbp | 35.72% |
| safe_skew_art_sbp | 35.72% |
| safe_trend_art_sbp | 35.72% |
| energy_art_dbp | 35.68% |
| max_art_dbp | 35.68% |
| mean_art_dbp | 35.68% |
| min_art_dbp | 35.68% |
| safe_entropy_art_dbp | 35.68% |
| safe_kurtosis_art_dbp | 35.68% |
| safe_skew_art_dbp | 35.68% |
| safe_trend_art_dbp | 35.68% |
| sum_eph | 33.41% |
| sum_ftn | 22.75% |
| mean_rfti | 18.53% |
| mean_ppfi | 16.20% |
| energy_cvp | 10.17% |
| max_cvp | 10.17% |
| mean_cvp | 10.17% |
| min_cvp | 10.17% |
| safe_kurtosis_cvp | 10.17% |
| safe_skew_cvp | 10.17% |
| safe_trend_cvp | 10.17% |
| sum_rbc | 5.49% |
| mean_ci | 4.41% |
| safe_entropy_cvp | 3.71% |
| sum_mdz | 2.46% |
| sum_ffp | 1.12% |
| sum_n2o | 1.05% |
| mean_ntgi | 0.49% |
| energy_pap_mbp | 0.44% |
| max_pap_mbp | 0.44% |
| mean_pap_mbp | 0.44% |
| min_pap_mbp | 0.44% |
| safe_entropy_pap_mbp | 0.44% |
| safe_kurtosis_pap_mbp | 0.44% |
| safe_skew_pap_mbp | 0.44% |
| safe_trend_pap_mbp | 0.44% |
| energy_pap_sbp | 0.41% |
| max_pap_sbp | 0.41% |
| mean_pap_sbp | 0.41% |
| min_pap_sbp | 0.41% |
| safe_entropy_pap_sbp | 0.41% |
| safe_kurtosis_pap_sbp | 0.41% |
| safe_skew_pap_sbp | 0.41% |
| safe_trend_pap_sbp | 0.41% |
| energy_pap_dbp | 0.40% |
| max_pap_dbp | 0.40% |
| mean_pap_dbp | 0.40% |
| min_pap_dbp | 0.40% |
| safe_entropy_pap_dbp | 0.40% |
| safe_kurtosis_pap_dbp | 0.40% |
| safe_skew_pap_dbp | 0.40% |
| safe_trend_pap_dbp | 0.40% |
| sum_pc | 0.34% |
| mean_mlni | 0.09% |
| mean_dobui | 0.09% |
| sum_cryo | 0.08% |
| mean_cbro2 | 0.07% |
| sum_sft | 0.05% |
| sum_pheresis | 0.04% |

## Supplemental File 6: Adapted GS-AKI Definition and Incidence

We implemented an adapted General Surgery Acute Kidney Injury (GS-AKI) comparator as a deterministic preoperative ordinal baseline. The score was not refit, recalibrated, or optimized in INSPIRE. Age, sex, emergency status, and preoperative creatinine were taken from the retained operation-level cohort. Diabetes, hypertension, congestive heart failure, and ascites were derived from diagnosis records documented before operation start; congestive heart failure and ascites were restricted to diagnoses within 30 days before surgery. Because INSPIRE does not provide a native intraperitoneal-surgery variable, intraperitoneal surgery was approximated using a committed 5-character ICD-10-PCS proxy map derived from CDC/NHSN operative-category mappings and CMS ICD-10-PCS order-file review, with explicit expert-reviewed overrides for residual observed code families.

The adapted score used nine grouped binary factors. The raw count was used as the ordinal prediction score for discrimination. Published simplified GS-AKI class cutpoints were applied as follows: Class I, 0-2 factors; Class II, 3 factors; Class III, 4 factors; Class IV, 5 factors; and Class V, 6-9 factors. Class III or higher, corresponding to a count of at least 4, was treated as the prespecified high-risk threshold.

| **Adapted GS-AKI factor** | **INSPIRE implementation** |
| --- | --- |
| Age >=56 years | Age at operation >=56 years |
| Male sex | Sex coded as male |
| Emergency surgery | Emergency operation indicator (emop == 1) |
| Intraperitoneal surgery | 5-character ICD-10-PCS intraperitoneal proxy map |
| Diabetes | Any preoperative diagnosis prefix E08, E09, E10, E11, or E13 |
| Congestive heart failure | Diagnosis prefix I50 within 30 days before operation start |
| Ascites | Diagnosis prefix R18 within 30 days before operation start |
| Hypertension | Any preoperative diagnosis prefix I10, I11, I12, I13, I15, I16, or I1A |
| Renal insufficiency | Preoperative creatinine >=1.2 mg/dL |

Held-out operation-level incidence increased monotonically across adapted GS-AKI scores and classes.

| **Score type** | **Score/class** | **Operations** | **Stage 2/3 AKI events** | **Event rate** | **95% CI** |
| --- | --- | --- | --- | --- | --- |
| Count | 0 | 7,408 | 39 | 0.005 | (0.004, 0.007) |
| Count | 1 | 20,292 | 268 | 0.013 | (0.012, 0.015) |
| Count | 2 | 17,806 | 497 | 0.028 | (0.026, 0.030) |
| Count | 3 | 8,699 | 438 | 0.050 | (0.046, 0.055) |
| Count | 4 | 2,280 | 266 | 0.117 | (0.104, 0.131) |
| Count | 5 | 497 | 90 | 0.181 | (0.148, 0.218) |
| Count | 6 | 63 | 15 | 0.238 | (0.140, 0.362) |
| Count | 7 | 10 | 4 | 0.400 | (0.122, 0.738) |
| Class | I | 45,506 | 804 | 0.018 | (0.016, 0.019) |
| Class | II | 8,699 | 438 | 0.050 | (0.046, 0.055) |
| Class | III | 2,280 | 266 | 0.117 | (0.104, 0.131) |
| Class | IV | 497 | 90 | 0.181 | (0.148, 0.218) |
| Class | V | 73 | 19 | 0.260 | (0.165, 0.376) |

##

## Supplemental File 7: Included Variables and Feature Extraction Strategy

| Feature Type | Features | Preprocessing |
| --- | --- | --- |
| Patient/Operation Characteristics | | |
| Continuous | Age, Height, Weight, ASA, Body surface area, Body mass index, Booking case length, Number of cardiac events prior to surgery,  Operation length, Anesthesia type | Normalized using z score |
| Categorical | Sex, Emergency Operation, Department Type | One hot encoding |
| Continuous Preoperative Lab Values | Total protein, Sodium, Potassium, Platelet count, Glucose, White blood cell count, Alanine transaminase, Chloride, Lymphocyte percentage, Phosphorus, Albumin, Fibrinogen, Creatinine, Prothrombin time, Total bilirubin, Alkaline phosphatase, Activated partial thromboplastin time, Calcium, Blood urea nitrogen, Aspartate transaminase, High-sensitive CRP, Hemoglobin, Hematocrit, Segmented neutrophil percentage | Normalized using z score |
| Continuous Intraoperative Values Collected Every 5 Minutes | Respiration rate, Heart rate, Peripheral oxygen saturation, Fraction of inspired oxygen, Mean airway pressure, End tidal concentration of carbon dioxide, Positive end expiratory pressure, Peak inspiratory pressure, Arterial mean blood pressure, calculated static lung compliance, Tidal volume, Arterial systolic blood pressure, Arterial diastolic blood pressure, Minute ventilation, Plateau pressure, Body temperature, End tidal concentration of anesthetic agent, Central venous pressure | Time-invariant: statistical feature extraction followed by z score normalization.  Time-variant: passed directly |
| Continuous Intraoperative Values Collected Every 10 Minutes | Pulmonary artery mean blood pressure, Pulmonary artery systolic blood pressure, Pulmonary artery diastolic blood pressure, Non-invasive mean blood pressure, Non-invasive diastolic blood pressure, Non-invasive systolic blood pressure | Time-invariant: statistical feature extraction followed by z score normalization.  Time-variant: passed directly |
| Averaged Continuous Intraoperative Variables | Flow of medical air, Bispectral index, cerebral regional oxygen saturation, Cardiac Index, Rate of dobutamine continuous infusion, Rate of milrinone continuous infusion, Rate of nitroglycerin continuous infusion, Flow of oxygen, Target concentration of propofol, Target concentration of remifentanil | Averaged over the whole operation |
| Summed Continuous Intraoperative Variables | Injected dose of Ephedrine, Injected dose of midazolam, Injected dose of propofol, Injected dose of sufentanil, Transfused cryoprecipitate, Estimated blood loss, Transfused fresh frozen plasma, Injected dose of fentanyl, Flow of nitrous oxide, Transfused platelet concentrate, Transfused platelet pheresis, Transfused red blood cell, Urine output, Summed Fluids, Equivalent MAC Totals | Summed  Fluids are an aggregate of all crystalloid fluids used.  Equiv_MAC_totals were calculated using MAC equivalents of different inhaled anesthetics |

##

## Supplemental File 8: Logistic Regression Variant Performance Investigation

| Regularization | Precision | Sensitivity | Accuracy | ROC AUC | PR AUC | Specificity | NPV | F1 Score | Balanced Accuracy | Training Time (s) |
| --- | --- | --- | --- | --- | --- | --- | --- | --- | --- | --- |
| None | 0.141596 | 0.689112 | 0.732634 | 0.788887 | 0.28183 | 0.73539 | 0.973921 | 0.234921 | 0.712251 | 11.115713 |
| L2 Ridge | 0.141384 | 0.687679 | 0.732634 | 0.789024 | 0.281974 | 0.735481 | 0.973808 | 0.234547 | 0.71158 | 10.494617 |
| L1 LASSO | 0.140684 | 0.689112 | 0.730756 | 0.789018 | 0.280214 | 0.733394 | 0.973852 | 0.233665 | 0.711253 | 29.247184 |
| ElasticNet | 0.140726 | 0.689112 | 0.730841 | 0.788991 | 0.280159 | 0.733485 | 0.973855 | 0.233722 | 0.711298 | 29.262273 |
| Regularization | Precision | Sensitivity | Accuracy | ROC AUC | PR AUC | Specificity | NPV (Class 0) | F1 Score | Balanced Accuracy | Training Time (s) |
| None | 0.197046 | 0.783668 | 0.796911 | 0.860824 | 0.264441 | 0.79775 | 0.983115 | 0.314911 | 0.790709 | 4.913799 |
| L2 Ridge | 0.196686 | 0.782235 | 0.79674 | 0.860796 | 0.264462 | 0.797659 | 0.983003 | 0.314335 | 0.789947 | 4.502826 |
| L1 LASSO | 0.19573 | 0.787966 | 0.794522 | 0.859565 | 0.266779 | 0.794937 | 0.983388 | 0.313569 | 0.791451 | 4.697007 |
| ElasticNet | 0.19573 | 0.787966 | 0.794522 | 0.859558 | 0.266795 | 0.794937 | 0.983388 | 0.313569 | 0.791451 | 4.756447 |
| Regularization | Precision | Sensitivity | Accuracy | ROC AUC | PR AUC | Specificity | NPV | F1 Score | Balanced Accuracy | Training Time (s) |
| None | 0.205927 | 0.776504 | 0.808329 | 0.875633 | 0.336836 | 0.810345 | 0.982831 | 0.325526 | 0.793425 | 17.335566 |
| L2 Ridge | 0.205682 | 0.777937 | 0.807817 | 0.875963 | 0.33715 | 0.80971 | 0.982926 | 0.325345 | 0.793823 | 14.327857 |
| L1 LASSO | 0.208775 | 0.790831 | 0.809012 | 0.876749 | 0.333165 | 0.810163 | 0.98391 | 0.330341 | 0.800497 | 32.699836 |
| ElasticNet | 0.208475 | 0.789398 | 0.808926 | 0.876728 | 0.333183 | 0.810163 | 0.983802 | 0.329841 | 0.799781 | 33.083192 |

##

## Supplemental File 9: Time-Variant Deep Learning Intraoperative Models Performance Metrics

| Model | Precision | Sensitivity | Accuracy | ROC AUC | PR AUC | Specificity | Negative Predictive Value | F1 Score |
| --- | --- | --- | --- | --- | --- | --- | --- | --- |
| RNN | 0.0851 | 0.8536 | 0.4406 | 0.7343 | 0.2178 | 0.4142 | 0.9779 | 0.1547 |
| LSTM | 0.0822 | 0.8675 | 0.4108 | 0.7409 | 0.2119 | 0.3817 | 0.9783 | 0.1501 |
| BILSTM | 0.083 | 0.8737 | 0.4132 | 0.7377 | 0.1967 | 0.3838 | 0.9794 | 0.1516 |
| SA-BILSTM | 0.0813 | 0.8737 | 0.4004 | 0.7368 | 0.2186 | 0.3701 | 0.9787 | 0.1488 |
| SESA-BILSTM | 0.0818 | 0.8644 | 0.4097 | 0.7326 | 0.2125 | 0.3807 | 0.9778 | 0.1494 |
| SEMA-2xBiLSTM | 0.0804 | 0.886 | 0.3854 | 0.7362 | 0.2156 | 0.3534 | 0.9798 | 0.1475 |
| Transformer | 0.0934 | 0.8043 | 0.5199 | 0.7467 | 0.2094 | 0.5017 | 0.9757 | 0.1674 |
| TCN | 0.0811 | 0.8921 | 0.387 | 0.7365 | 0.217 | 0.3548 | 0.981 | 0.1487 |

**RNN: Recurrent Neural Network; LSTM: Long Short-Term Memory; BiLSTM: Bidirectional Long Short-Term Memory; SA-BiLSTM: Single Head Attention w/ Bidirectional Long Short-Term Memory; SESA: Single Head Attention w/ Squeeze & Excitation Block w/ Bidirectional Long Short-Term Memory; SEMA-2xBiLSTM: Multihead Attention w/ Squeeze & Excitation Block w/ Stacked Bidirectional Long Short-Term Memory; TCN: Temporal Convolutional Network**

Each model was trained for up to 100 epochs with early stopping. Hyperparameter Optimization (HPO) was performed using Optuna with 50 trials with trial pruning. Imbalanced data was handled by modifying sample weights (pos_weight via BCEWithLogitsLoss) in PyTorch.

## Supplemental File 10: SEMA-Stacked BiLSTM Architecture


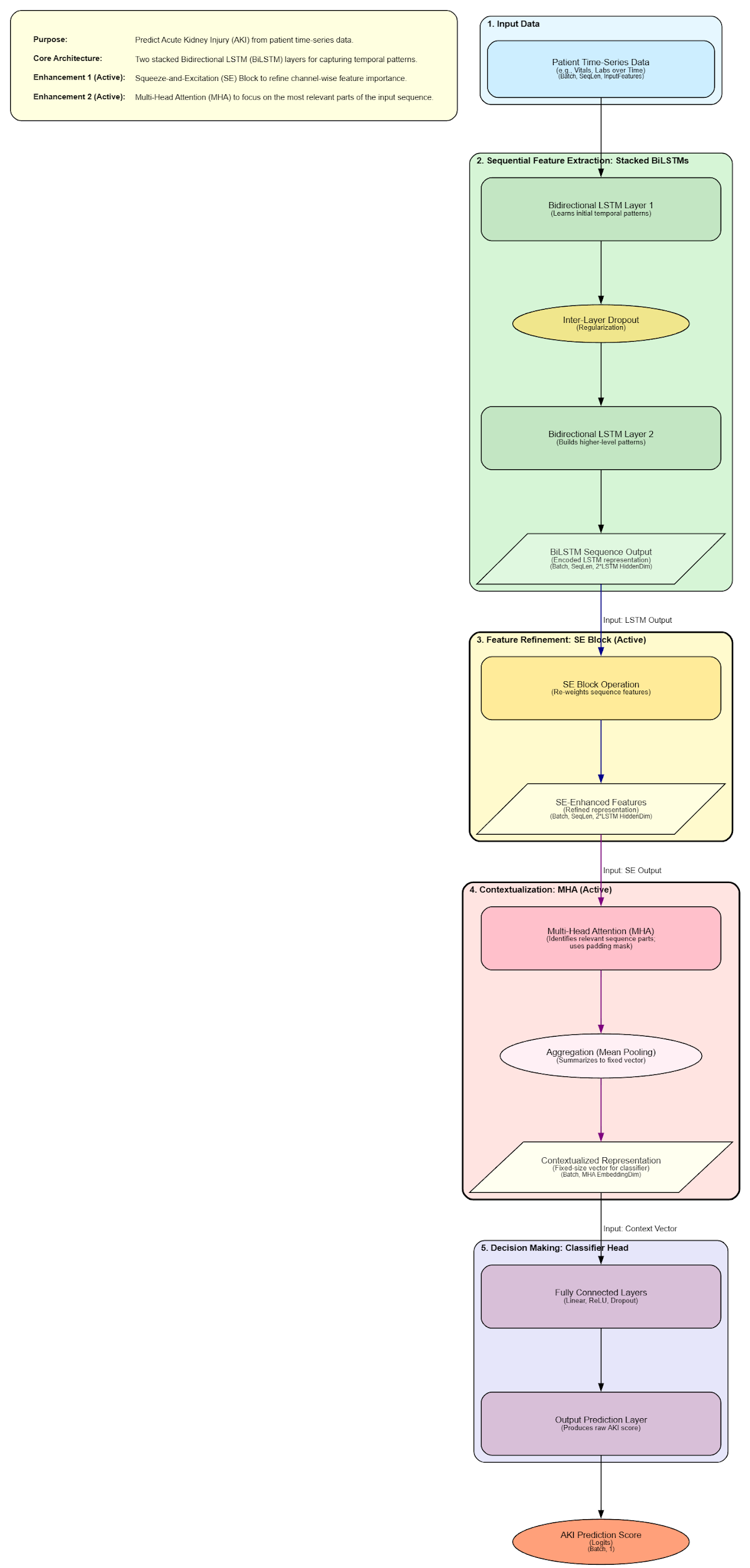


## Supplemental File 11: Transformer Architecture


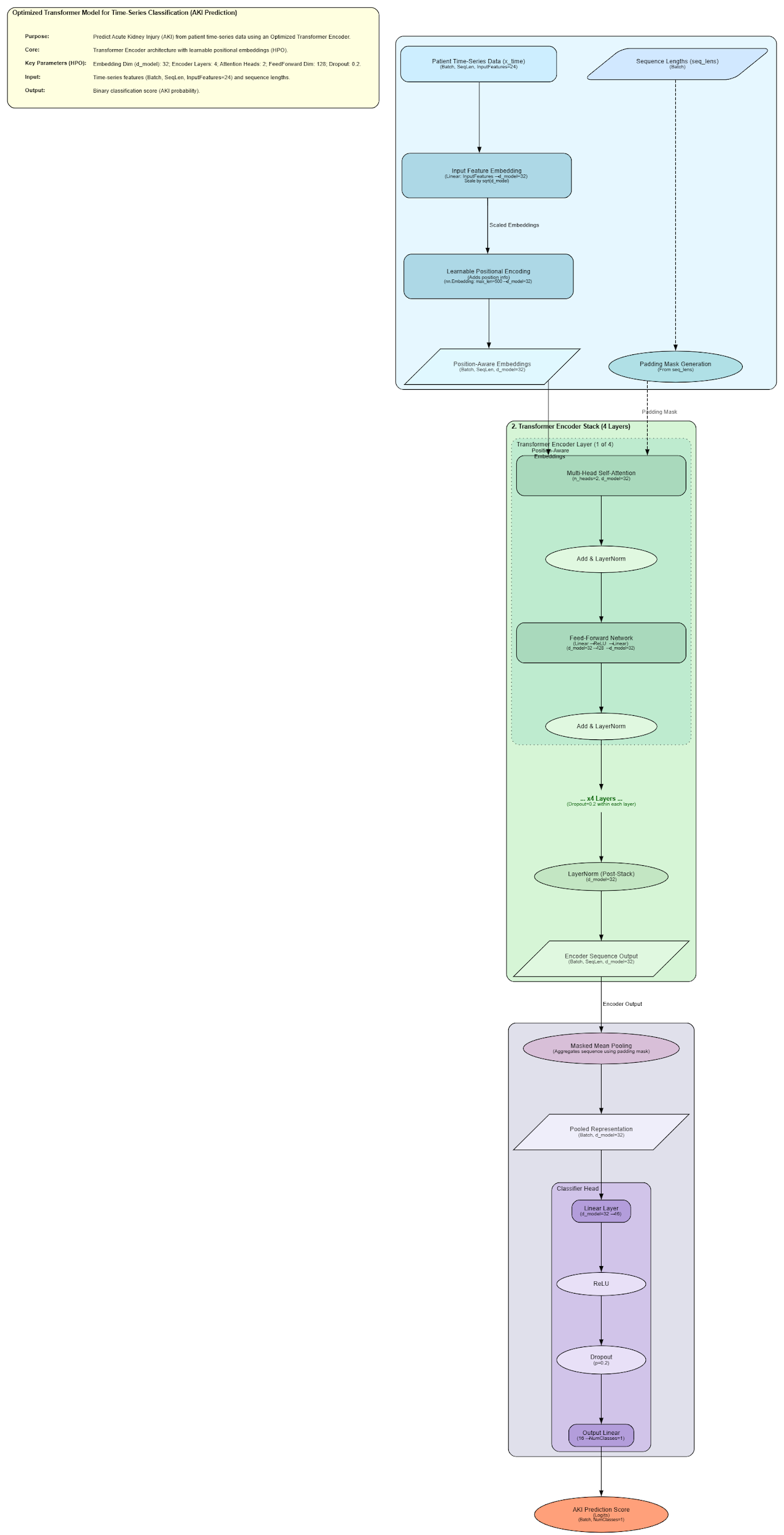


## Supplemental File 12: Temporal Convolutional Network (TCN) Architecture


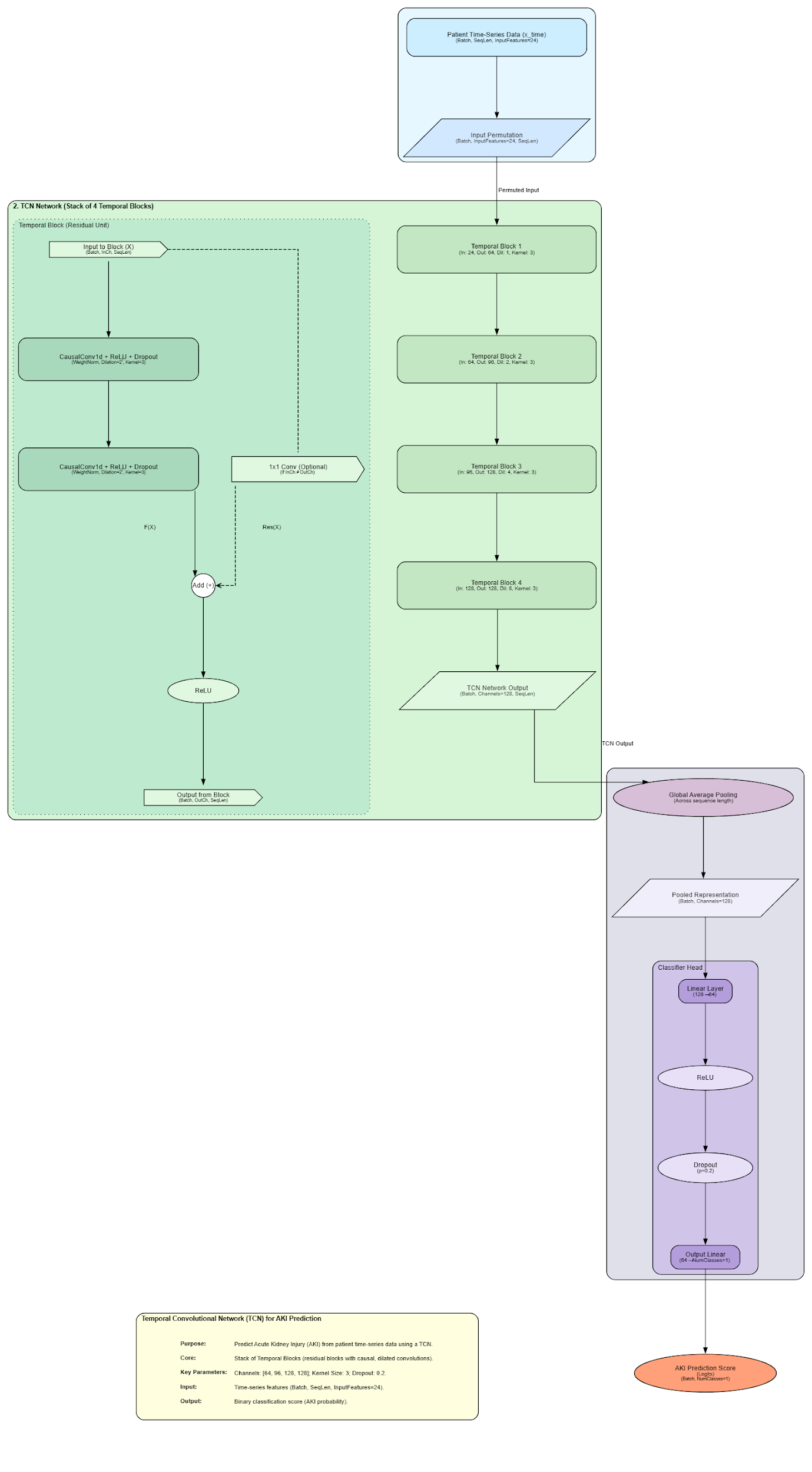


## Supplemental File 13: MLP + LSTM Model Architecture


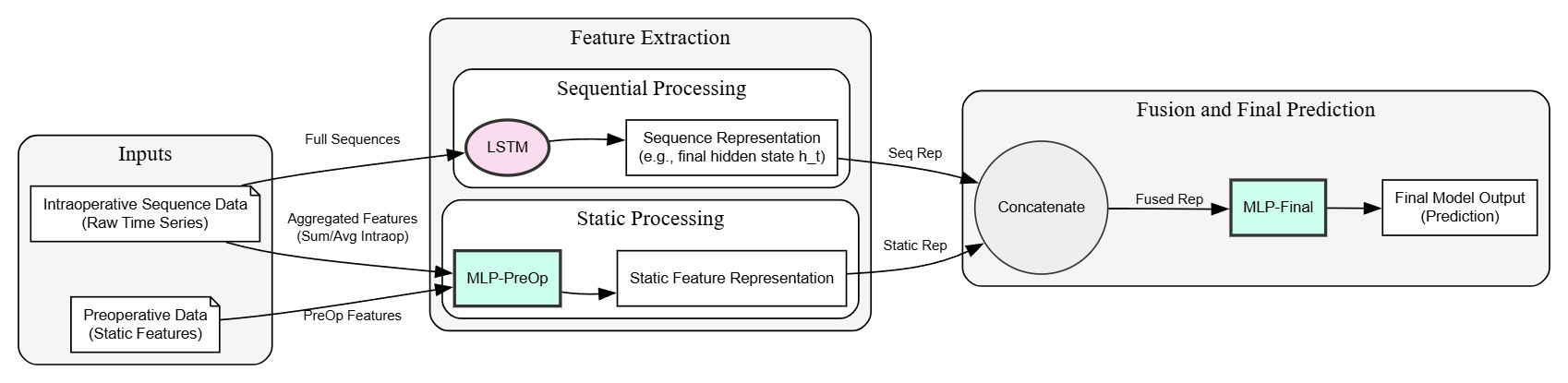


## Supplemental File 14: SHAP Beeswarm for Predictive Models

**Logistic Regression Combined
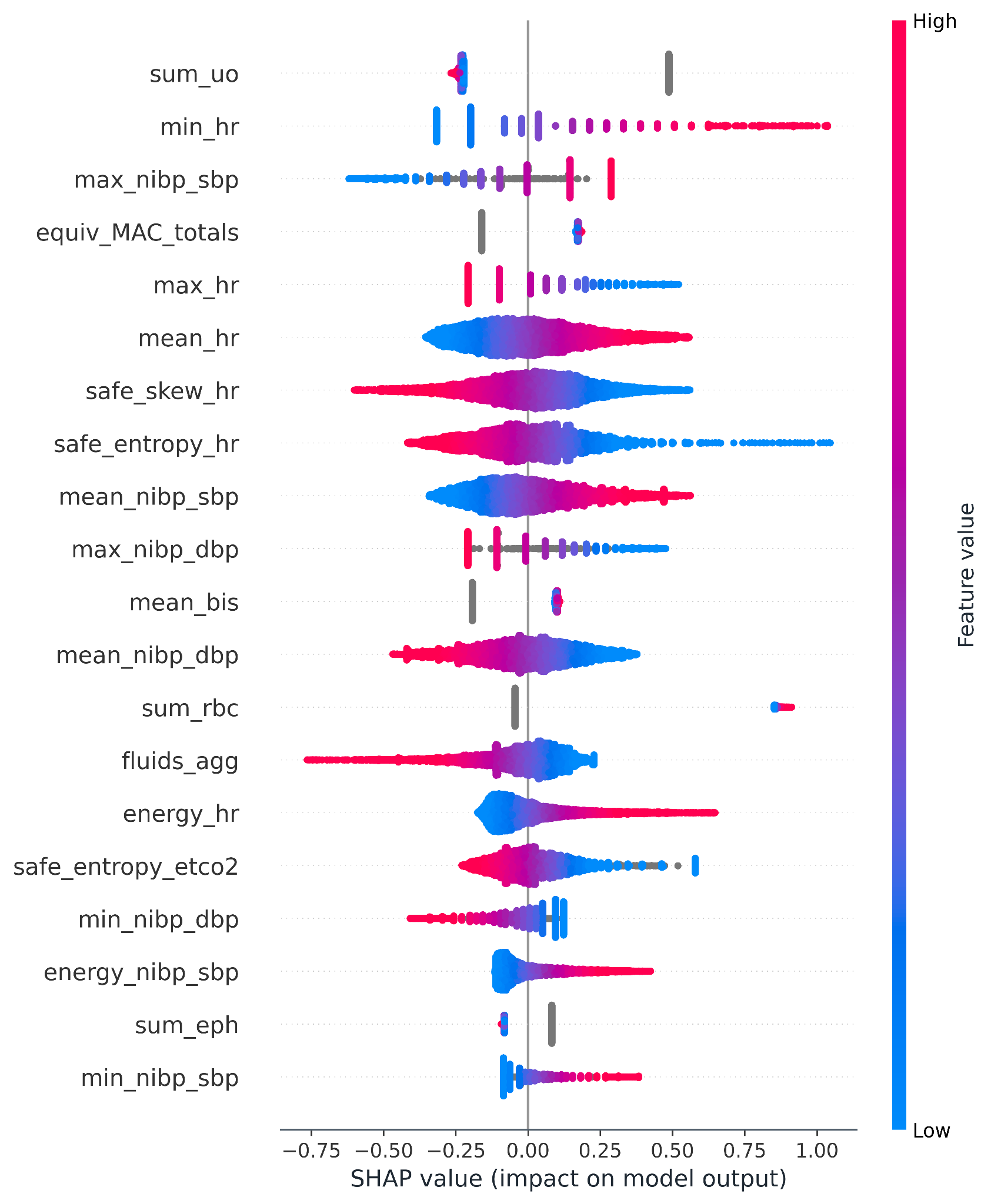
**

**Random Forest Combined
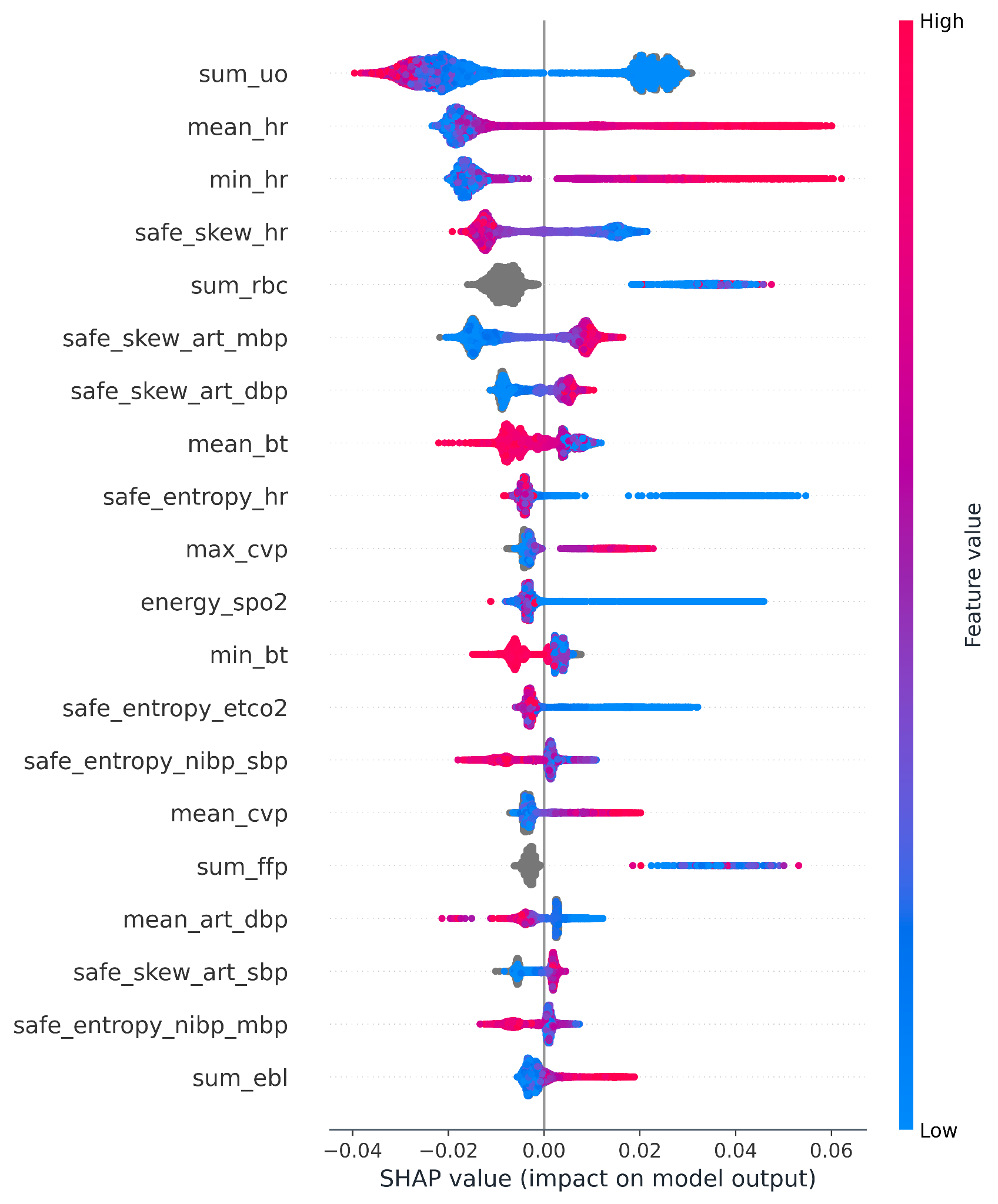
**

**Gradient Boosting Tree Combined
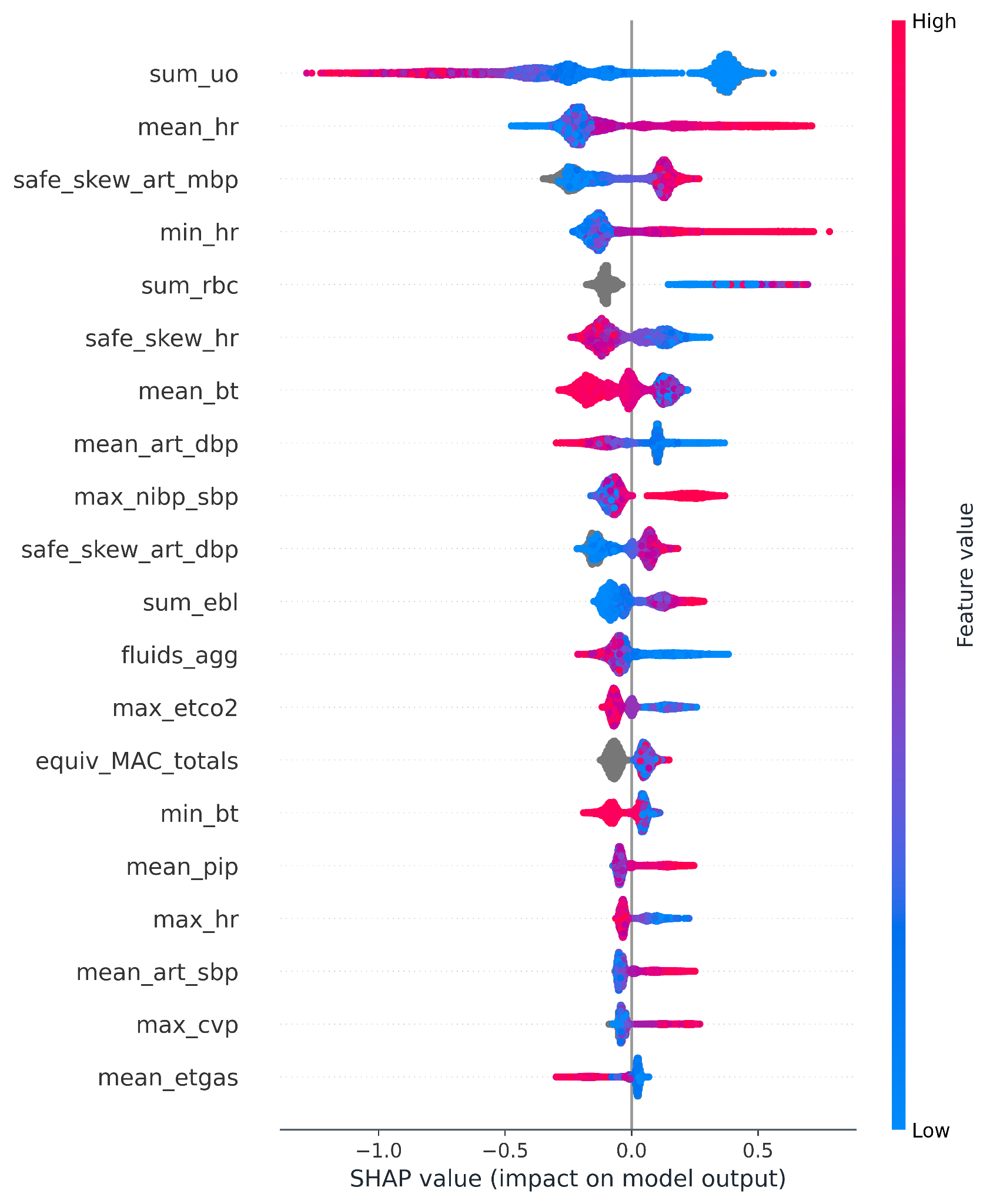
**

**Logistic Regression Intraoperative
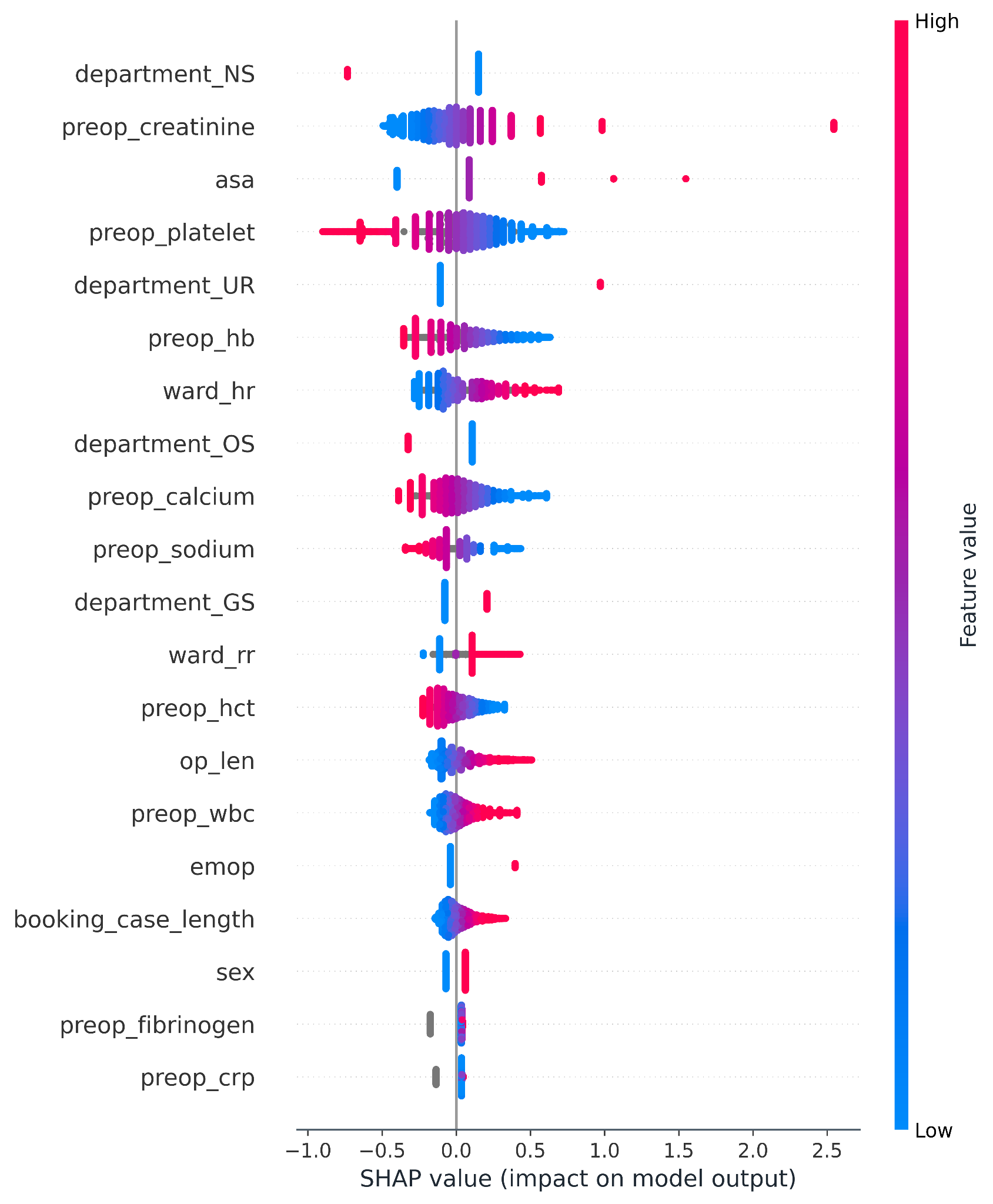
**

**Random Forest Intraoperative
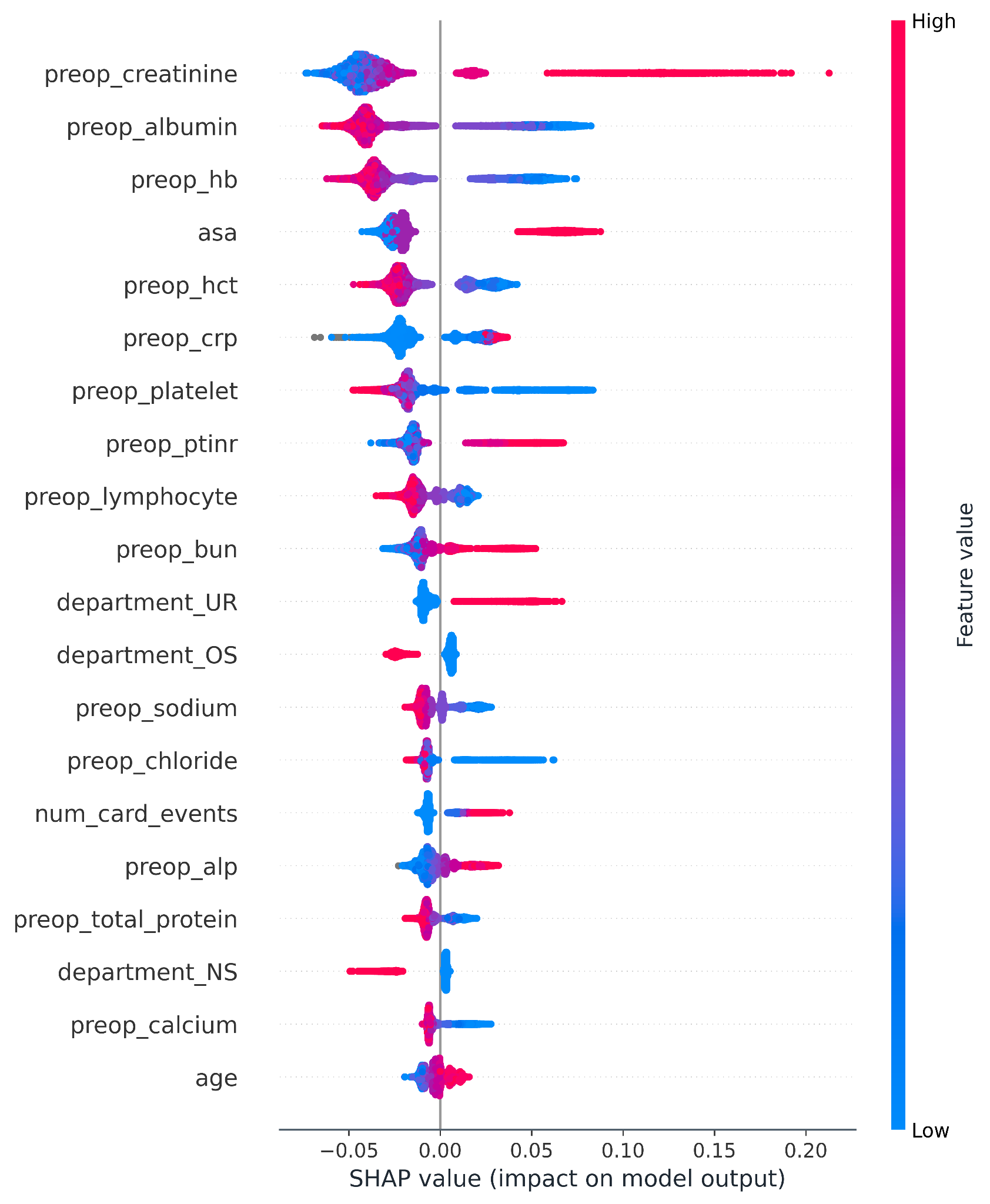
**

**Gradient Boosting Tree Intraoperative
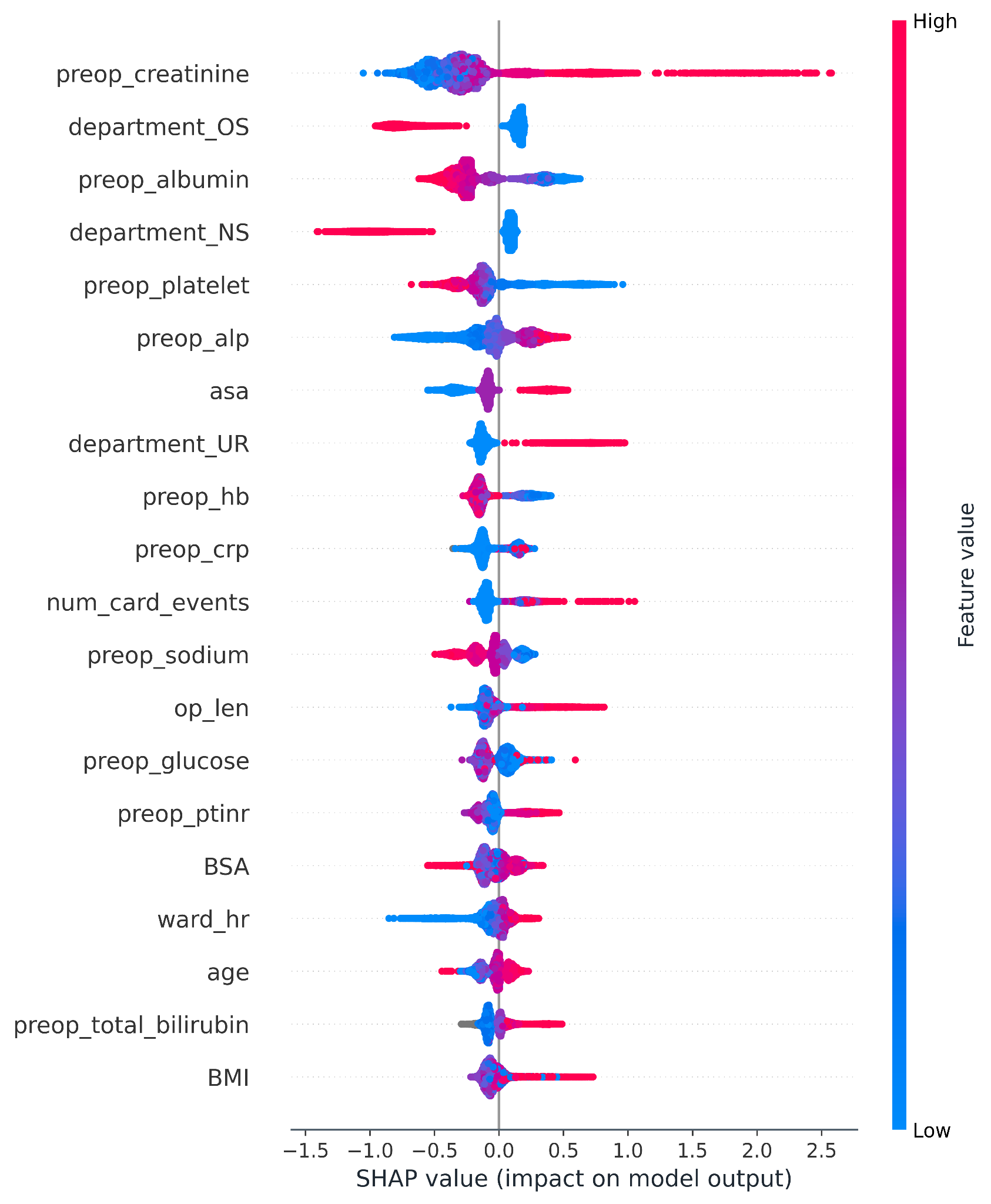
**

**Logistic Regression Preoperative
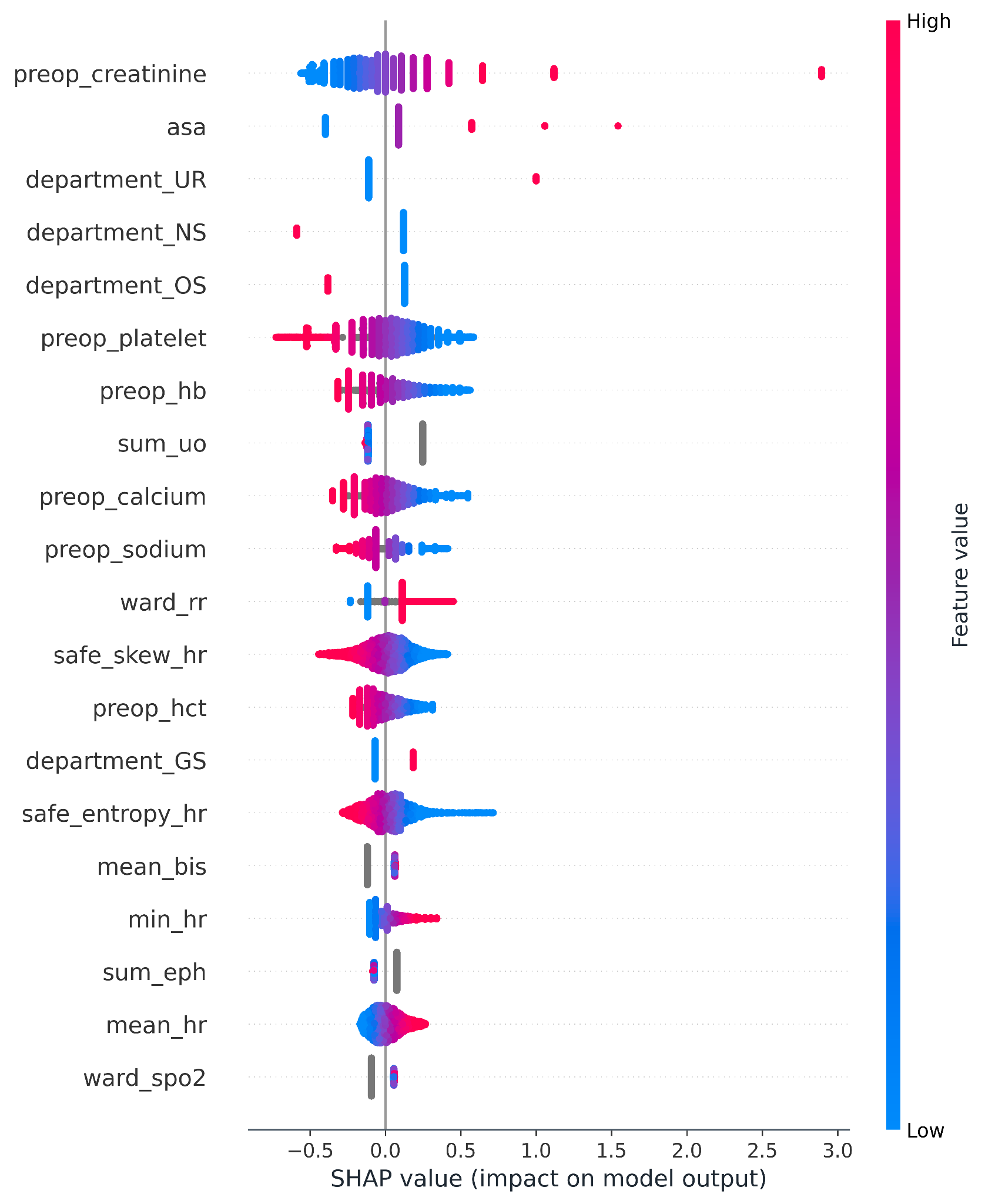
**

**Random Forest Preoperative
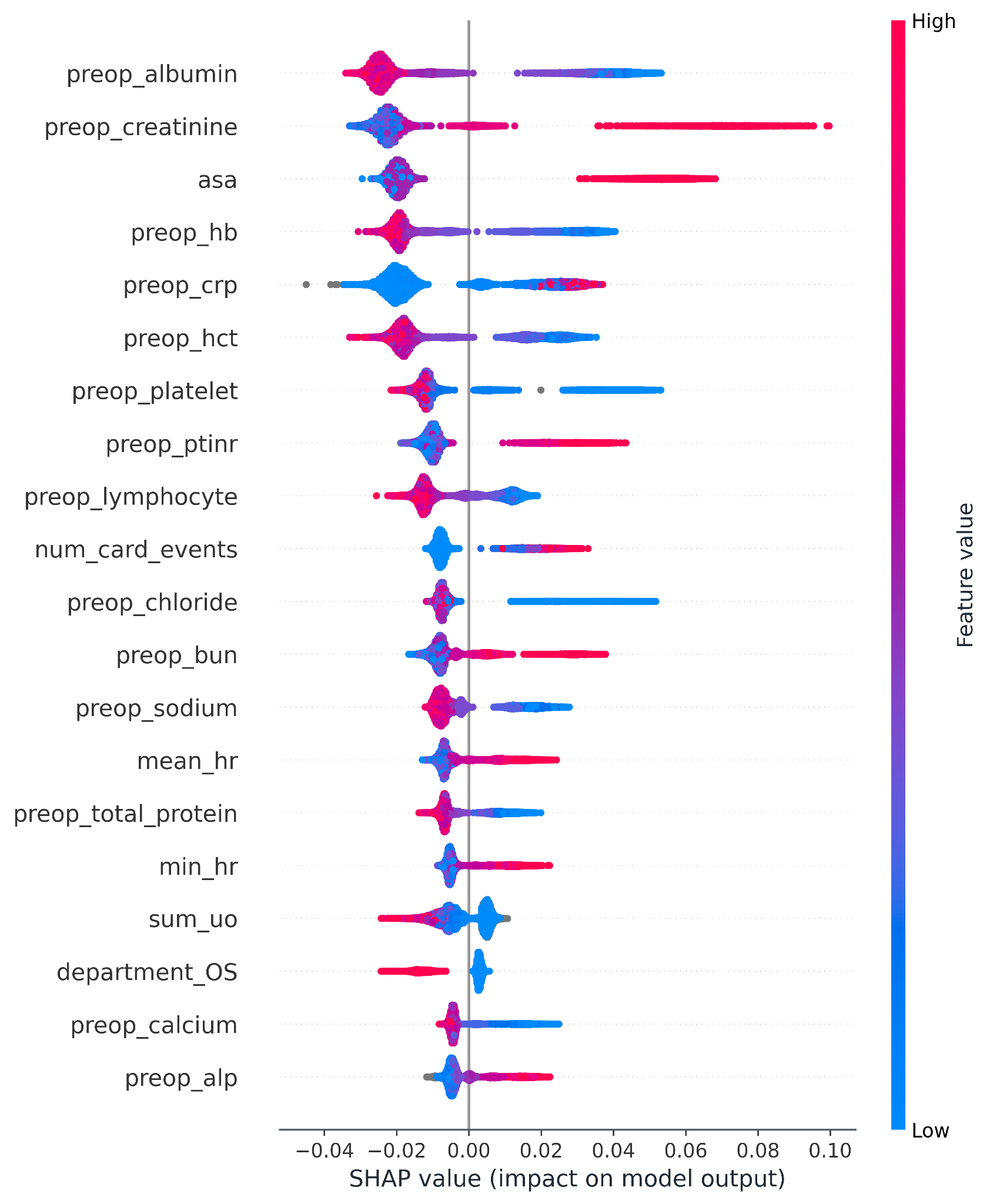
**

**Gradient Boosting Tree Preoperative
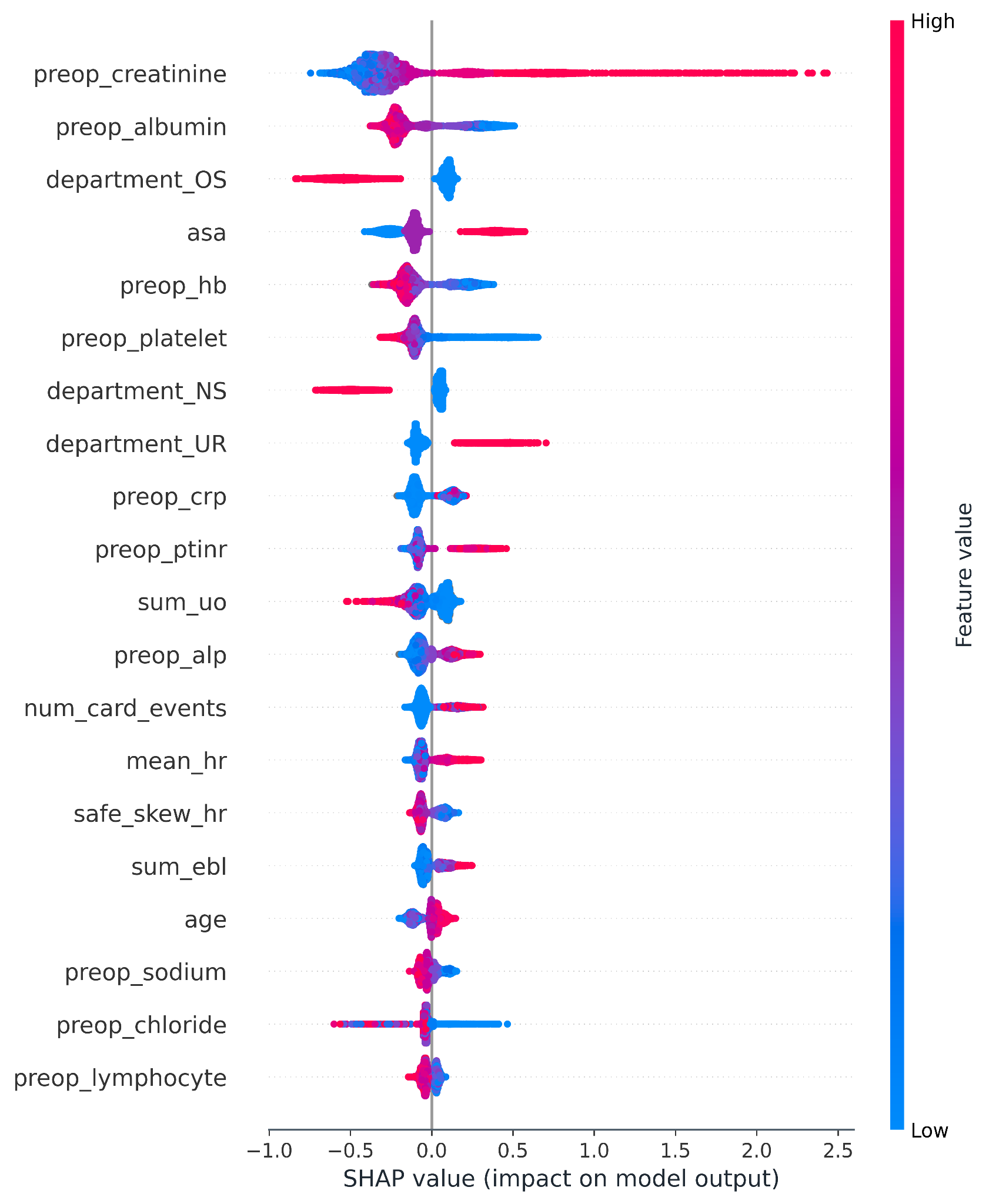
**

## Supplemental File 15: DeLong Pairwise Tests with Benjamini-Hochberg FDR Correction

|  | **c_AG** | **c_GBT** | **c_KNN** | **c_LR** | **c_MLP** | **c_RF** | **c_SVM** | **p_AG** | **p_GBT** | **p_KNN** | **p_LR** | **p_MLP** | **p_RF** | **p_SVM** |
| --- | --- | --- | --- | --- | --- | --- | --- | --- | --- | --- | --- | --- | --- | --- |
| **c_AG** | --- | **<0.0001***** | **<0.0001***** | **<0.0001***** | **<0.0001***** | **<0.0001***** | 0.1260 | **<0.0001***** | **<0.0001***** | **<0.0001***** | **<0.0001***** | **<0.0001***** | **<0.0001***** | 0.3194 |
| **c_GBT** |  |  | **<0.0001***** | **<0.0001***** | **<0.0001***** | **<0.0001***** | **<0.0001***** | **<0.0001***** | **<0.0001***** | **<0.0001***** | **<0.0001***** | **<0.0001***** | **<0.0001***** | **<0.0001***** |
| **c_KNN** |  |  | --- | 0.1799 | **0.0135*** | 0.5850 | **<0.0001***** | 0.2498 | **<0.0001***** | **0.0008***** | **0.0371*** | **0.0005***** | **0.0012**** | **0.0012**** |
| **c_LR** |  |  |  | --- | 0.1670 | 0.3892 | **<0.0001***** | 0.0705 | **0.0010**** | 0.2678 | 0.4113 | **0.0287*** | 0.2253 | **0.0005***** |
| **c_MLP** |  |  |  |  | --- | **0.0351*** | **<0.0001***** | **<0.0001***** | 0.0958 | 0.5384 | 0.5039 | 0.1096 | 0.6387 | **<0.0001***** |
| **c_RF** |  |  |  |  |  | --- | **<0.0001***** | 0.1812 | **<0.0001***** | **0.0141*** | 0.0996 | **0.0022**** | **0.0056**** | **0.0009***** |
| **c_SVM** |  |  |  |  |  |  | --- | **<0.0001***** | **<0.0001***** | **<0.0001***** | **<0.0001***** | **<0.0001***** | **<0.0001***** | **0.0032**** |
| **p_AG** |  |  |  |  |  |  |  | --- | **<0.0001***** | **0.0004***** | **0.0028**** | **<0.0001***** | **0.0004***** | **0.0050**** |
| **p_GBT** |  |  |  |  |  |  |  |  | --- | **0.0043**** | **0.0027**** | 0.2925 | **0.0072**** | **<0.0001***** |
| **p_KNN** |  |  |  |  |  |  |  |  |  | --- | 0.8117 | 0.0946 | 0.7083 | **<0.0001***** |
| **p_LR** |  |  |  |  |  |  |  |  |  |  | --- | 0.1184 | 0.6693 | **<0.0001***** |
| **p_MLP** |  |  |  |  |  |  |  |  |  |  |  | --- | 0.1430 | **<0.0001***** |
| **p_RF** |  |  |  |  |  |  |  |  |  |  |  |  | --- | **<0.0001***** |
| **p_SVM** |  |  |  |  |  |  |  |  |  |  |  |  |  | --- |

**Prefix: c: combined pre- and intraoperative training data. p: preoperative training data only. AG: AutoGluon, GBT: Gradient Boosting Tree; LR: Logistic Regression w/ Ridge; RF: Random Forest; MLP: Multilayer Perceptron; SVM: Support Vector Machine; KNN: K-Nearest Neighbors.**

## Supplemental File 15: AUPRC and Calibration Curves

| Data Type | Precision-Recall Curve | Calibration Curve |
| --- | --- | --- |
| Preoperative | 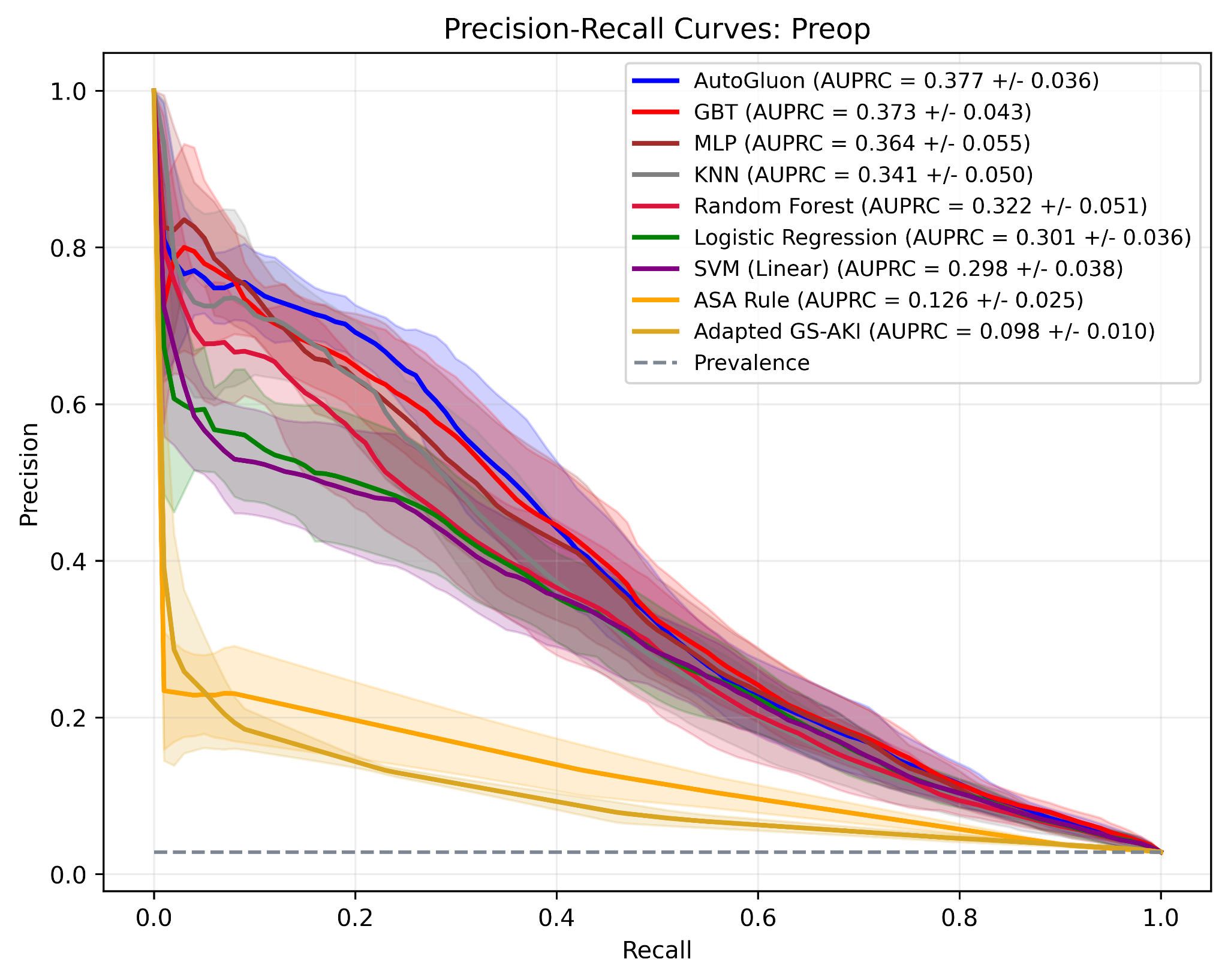 | 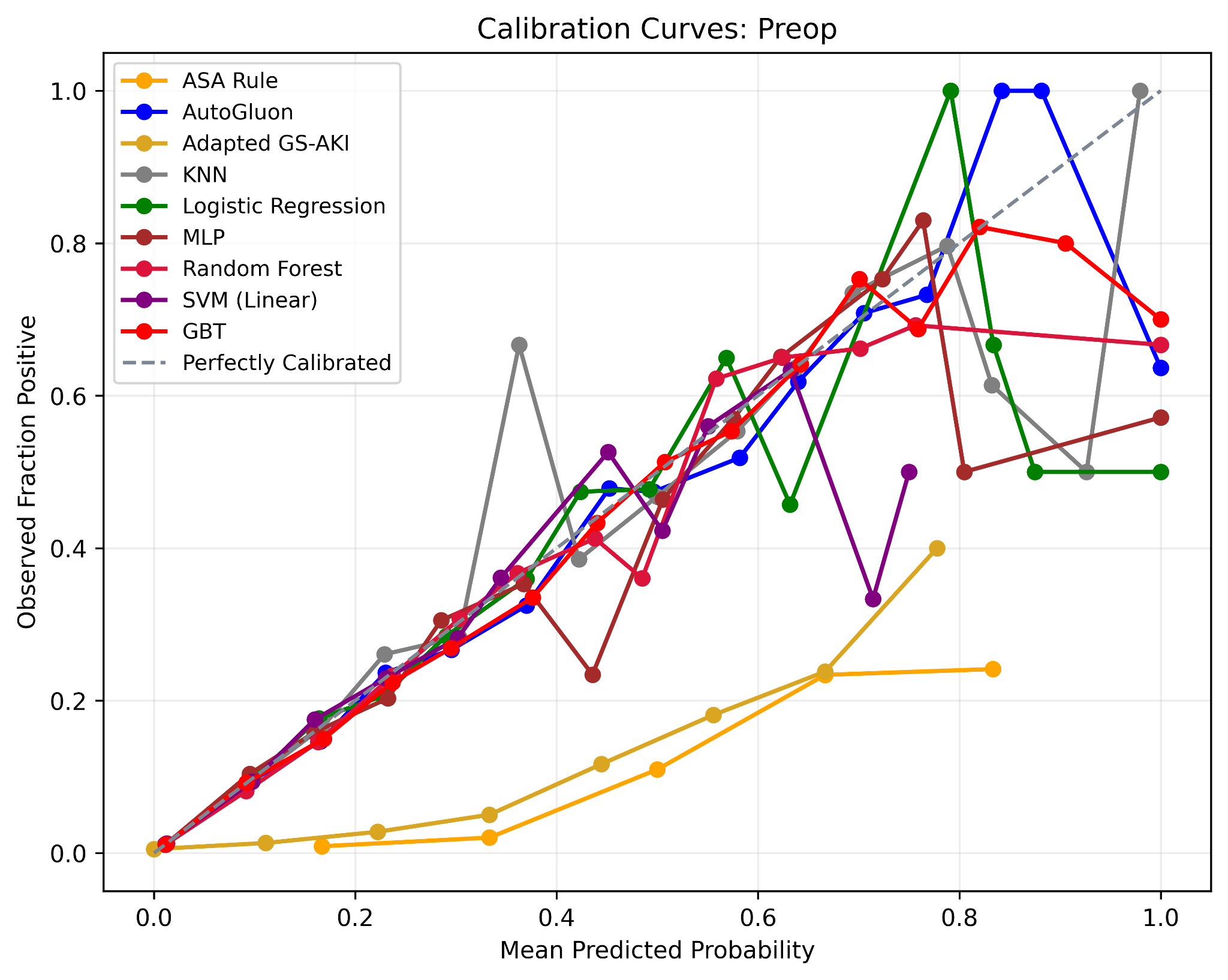 |
| Intraoperative | 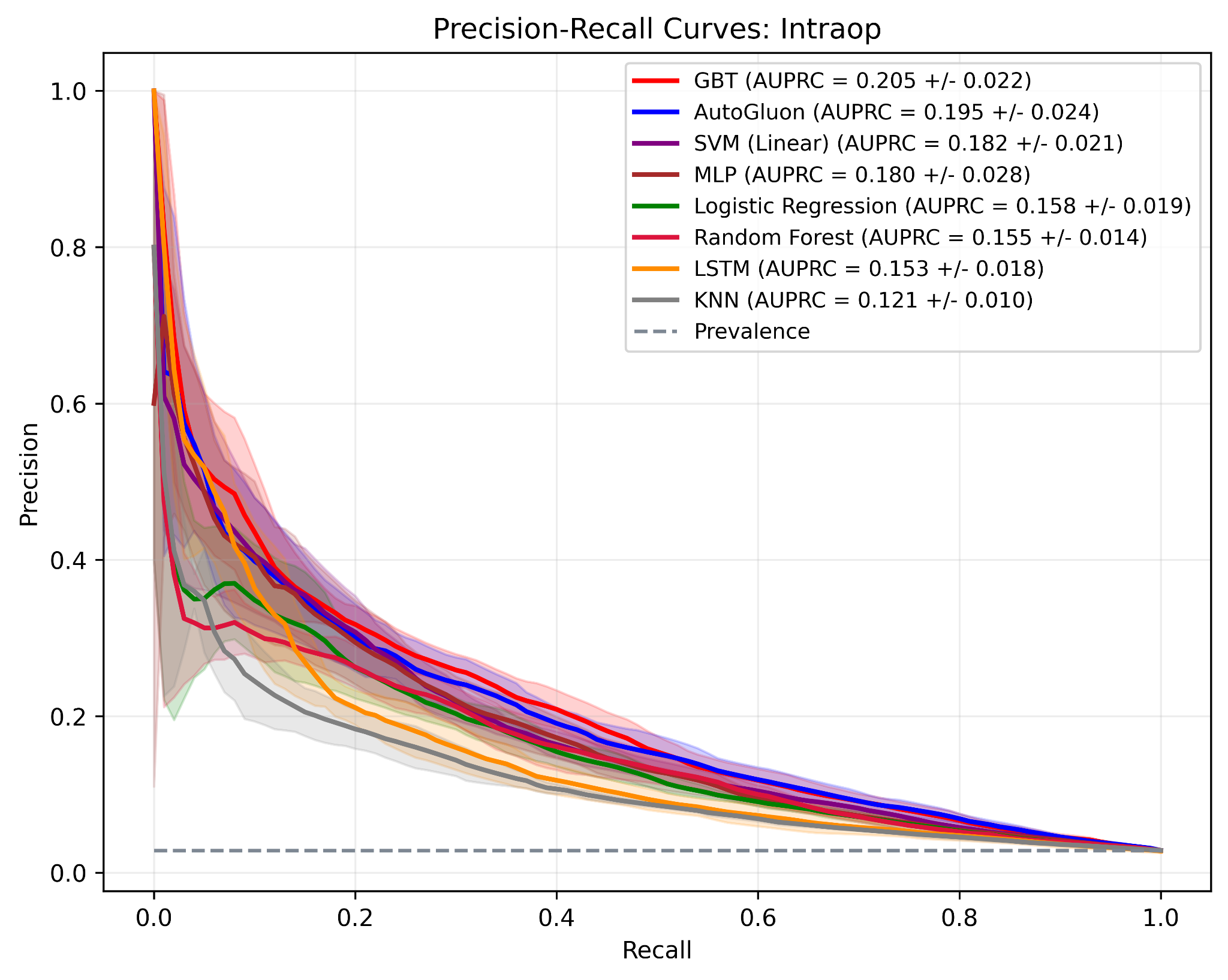 | 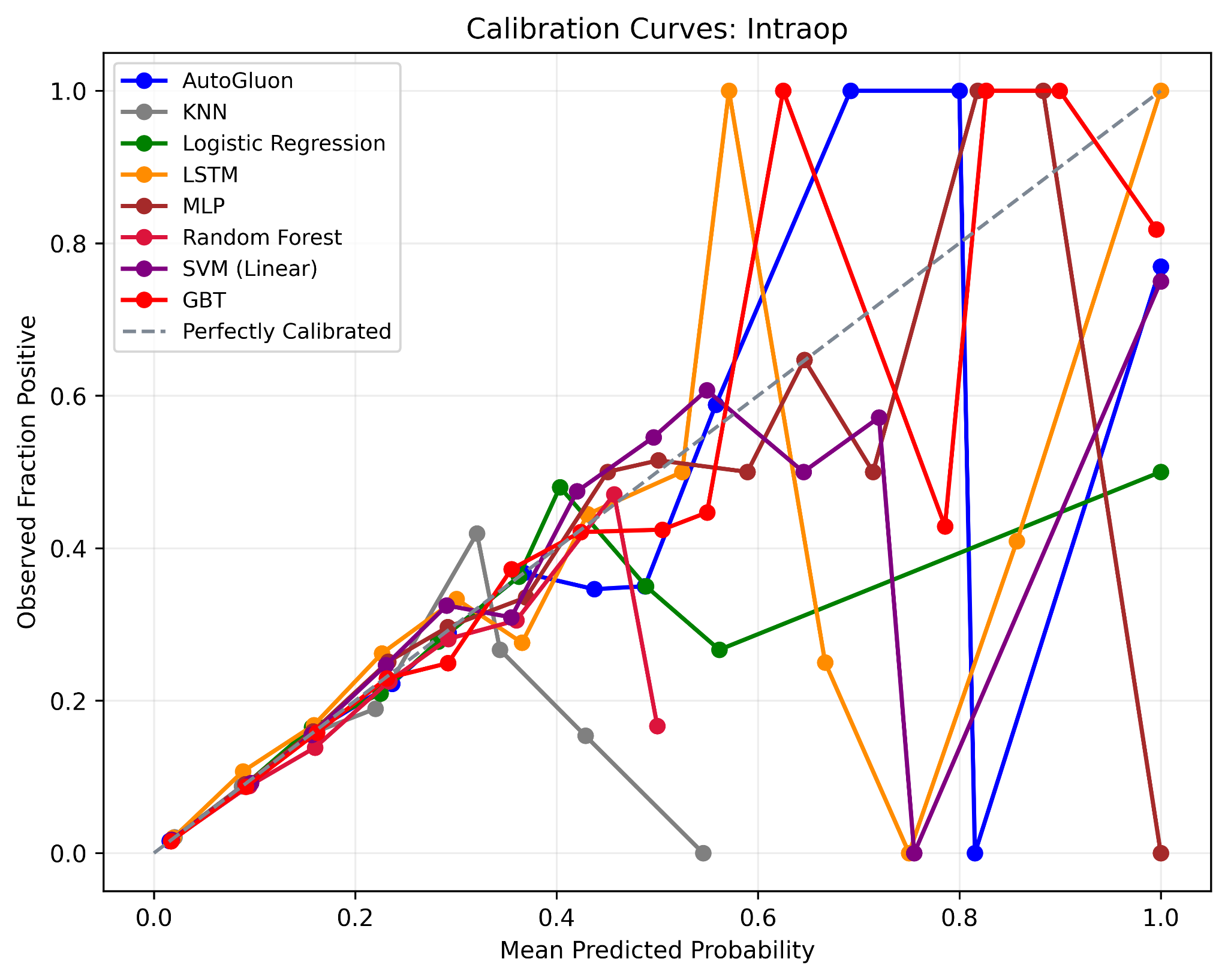 |
| Preoperative + Intraoperative | 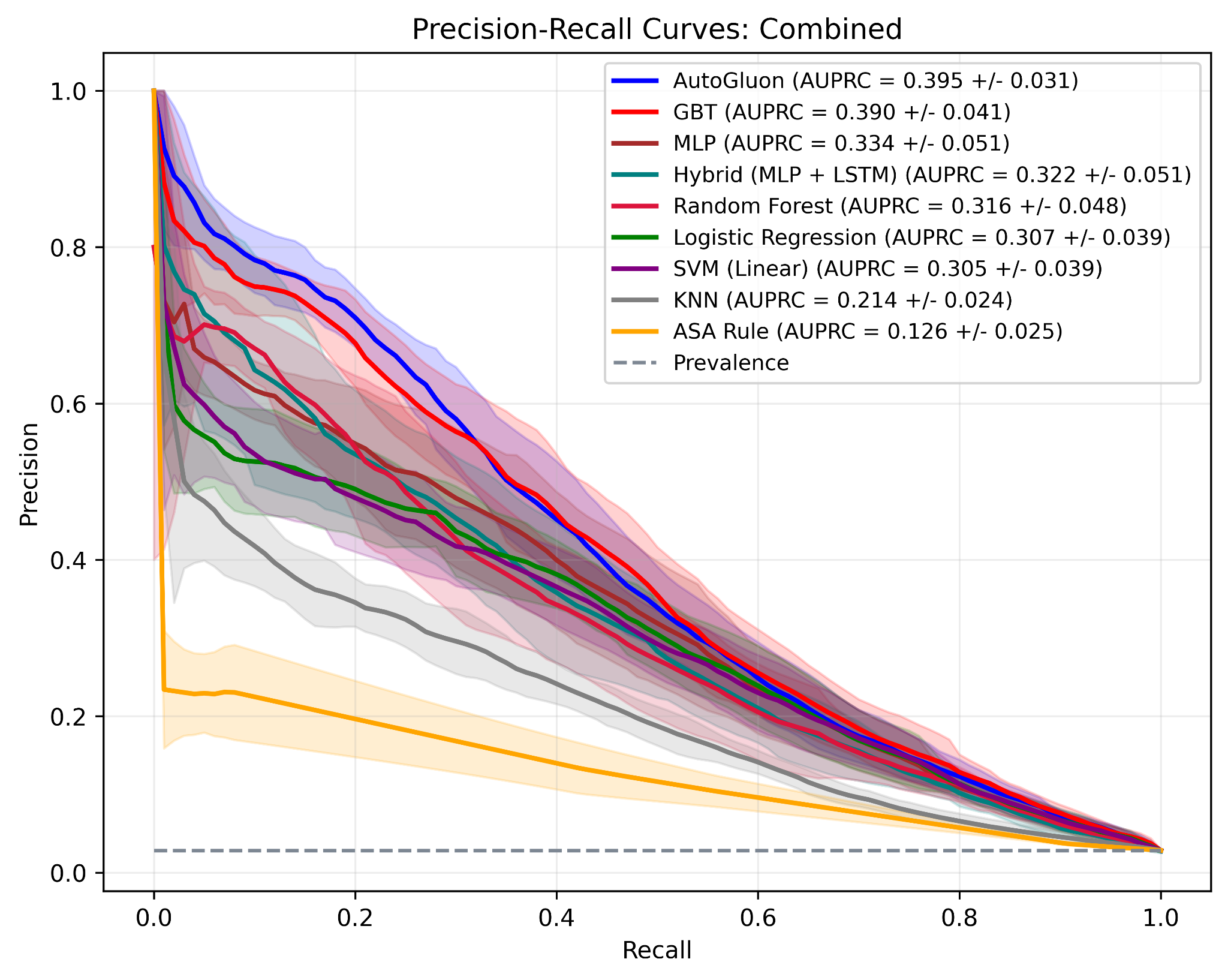 | 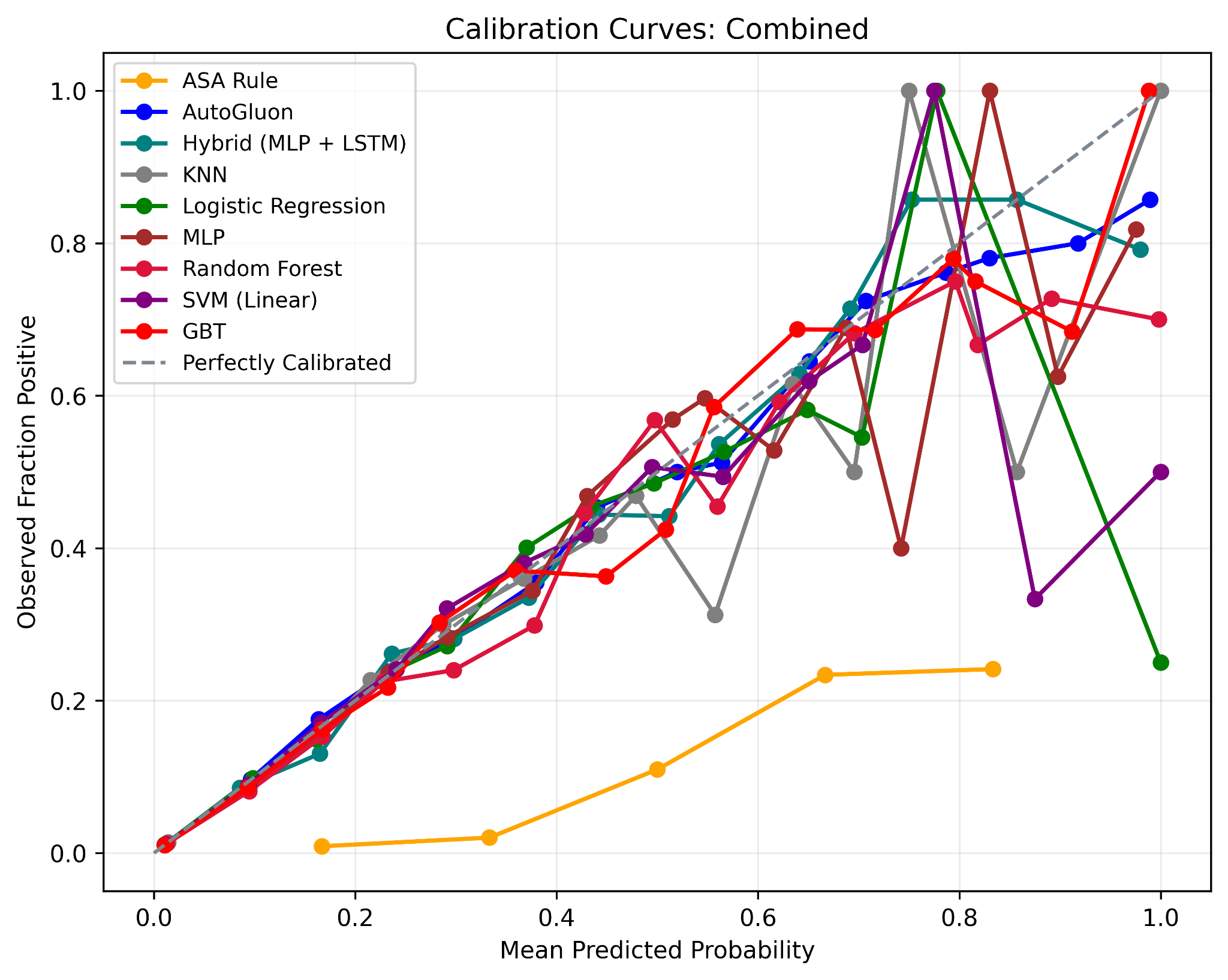 |

**ASA: American Society of Anesthesiology Physical Status Score Threshold ≥ 3; GBT: Gradient Boosting Tree; LR: Logistic Regression w/ Ridge; RF: Random Forest; MLP: Multilayer Perceptron; SVM: Support Vector Machine; KNN: K-Nearest Neighbors; MLP+LSTM: MLP (Preoperative) + Long Short-Term Memory (Time-Series Intraoperative) Hybrid Model**

## Supplemental File 16: Model Reclassification Analysis

| **Model** | **Correction Rate: Intraop -> Preop** | **Correction Rate: Preop -> Combined** |
| --- | --- | --- |
| ASA Rule | N/A | 0.0% (0.0 / 168.8) |
| AutoGluon | 36.6% (60.4 / 163.2) | 8.9% (12.4 / 138.4) |
| GBT | 35.2% (57.4 / 163.0) | 12.1% (17.6 / 144.0) |
| KNN | **44.8% (85.8 / 191.0)** | **22.8% (32.8 / 142.8)** |
| Logistic Regression | 43.9% (79.0 / 179.6) | 12.0% (16.8 / 130.8) |
| MLP | 39.4% (68.2 / 173.0) | 13.9% (20.0 / 139.8) |
| Random Forest | 38.0% (64.8 / 170.0) | 10.3% (15.6 / 147.8) |
| SVM (Linear) | 42.1% (76.8 / 182.0) | 10.7% (14.8 / 137.0) |

**Correction rates for each model when transitioning from a lower-feature to a higher-feature dataset. The rate represents the percentage of initially missed positive cases (false negatives) that were correctly reclassified. Values in parentheses indicate the average number of correctly reclassified patients out of the average number of initially missed positive cases per fold.**

## Supplemental File 17: Decision Curve Analysis for AutoGluon and GBT Models
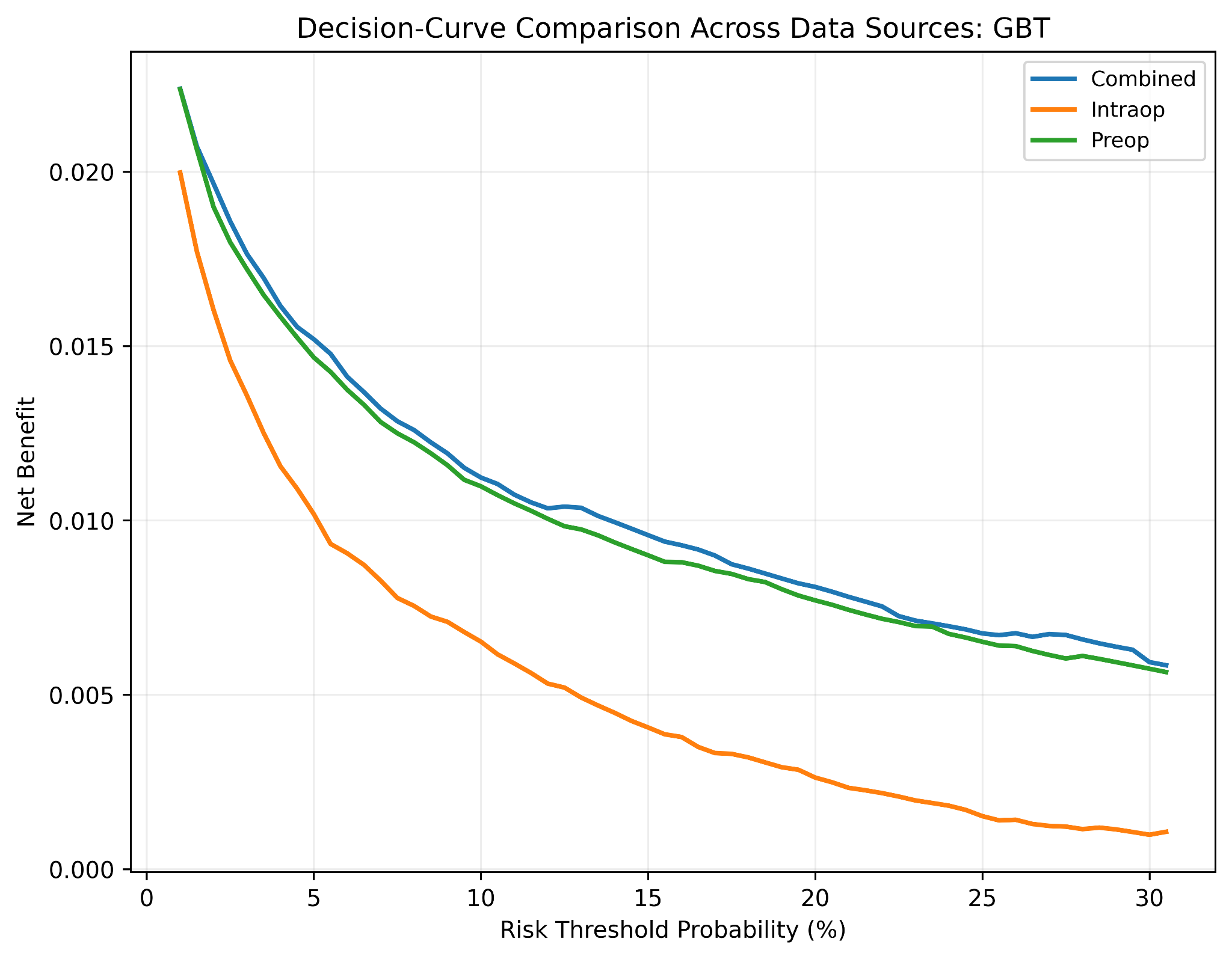

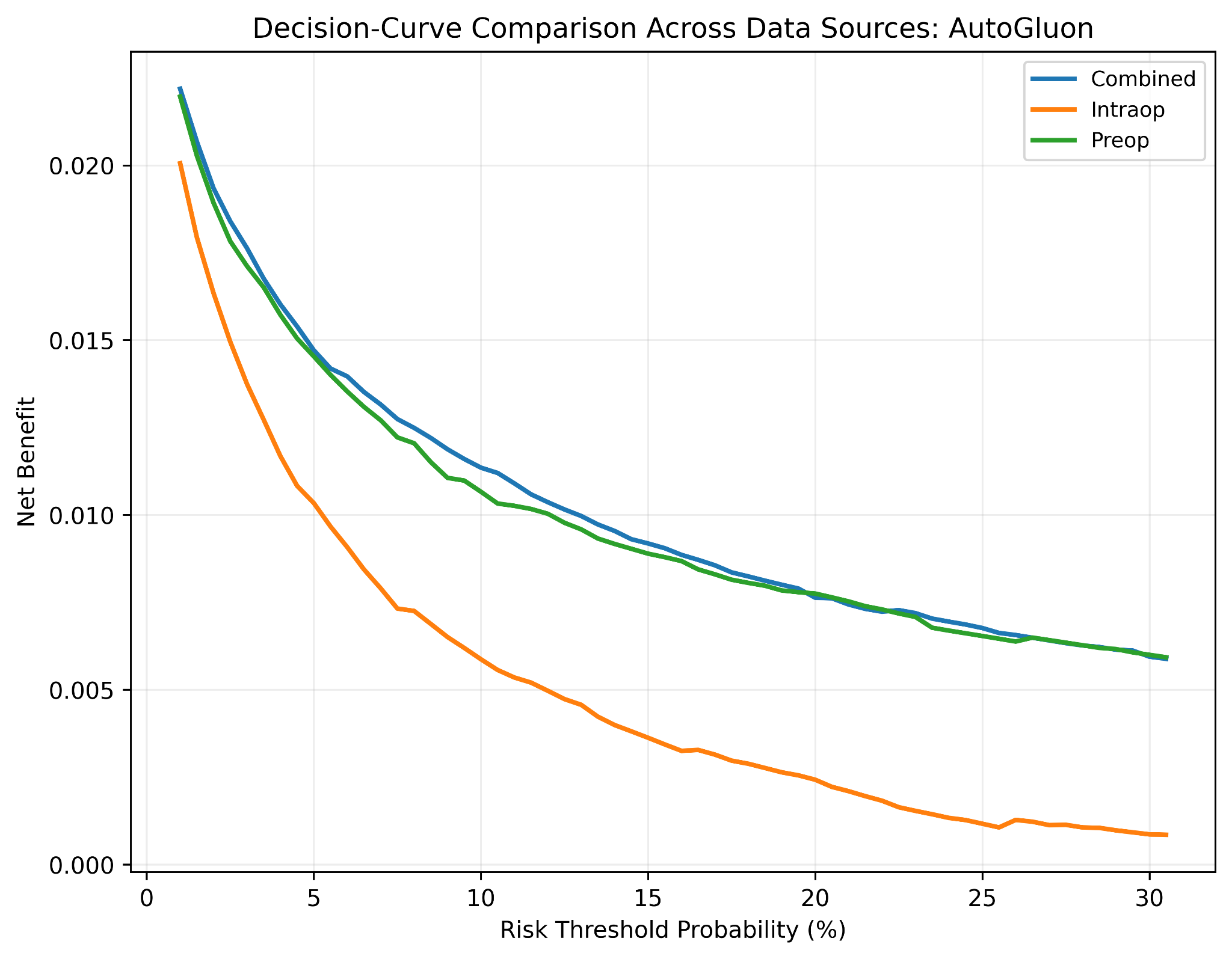

Supplement: ooag092_Supplementary_Data [file ooag092_supplementary_data.docx]
